# Development of three-layer collagen scaffolds to spatially direct tissue-specific cell differentiation for enthesis repair

**DOI:** 10.1016/j.mtbio.2023.100584

**Published:** 2023-03-07

**Authors:** Eugenia Pugliese, Ignacio Sallent, Sofia Ribeiro, Alexandre Trotier, Stefanie H. Korntner, Yves Bayon, Dimitrios I. Zeugolis

**Affiliations:** a Regenerative, Modular & Developmental Engineering Laboratory (REMODEL), University of Galway, Galway, Ireland; b Science Foundation Ireland (SFI) Centre for Research in Medical Devices (CÚRAM), University of Galway, Ireland; c Medtronic, Trevoux, France; d Regenerative, Modular & Developmental Engineering Laboratory (REMODEL), Charles Institute of Dermatology, Conway Institute of Biomolecular & Biomedical Research and School of Mechanical & Materials Engineering, University College Dublin (UCD), Dublin, Ireland

**Keywords:** Stem cell differentiation, Functionalisation, Bioactive molecules, Growth factors, Tendon-to-bone interface

## Abstract

Enthesis repair remains a challenging clinical indication. Herein, a three-layer scaffold composed of a tendon-like layer of collagen type I, a fibrocartilage-like layer of collagen type II and a bone-like layer of collagen type I and hydroxyapatite, was designed to recapitulate the matrix composition of the enthesis. To aid tenogenic and fibrochondrogenic differentiation, bioactive molecules were loaded in the tendon-like layer or the fibrocartilage-like layer and their effect was assessed in *in vitro* setting using human bone marrow derived mesenchymal stromal cells and in an *ex vivo* model. Seeded human bone marrow mesenchymal stromal cells infiltrated and homogeneously spread throughout the scaffold. As a response to the composition of the scaffold, cells differentiated in a localised manner towards the osteogenic lineage and, in combination with differentiation medium, towards the fibrocartilage lineage. Whilst functionalisation of the tendon-like layer did not improve tenogenic cell commitment within the time frame of this work, relevant fibrochondrogenic markers were detected in the fibrocartilage-like layer when scaffolds were functionalised with bone morphogenetic protein 2 or non-functionalised at all, *in vitro* and *ex vivo*, respectively. Altogether, our data advocate the use of compartmentalised scaffolds for the repair and regeneration of interfacial tissues, such as enthesis.

## Highlights

• A three-layer enthesis-composition inspired collagen scaffold was developed.
• The porous network of the tendon-like layer supported initial cellular alignment.
• The bone-like layer promoted local cell commitment towards osteogenic lineage.
• Growth factor functionalisation enhanced spatial fibrochondrogenic cell commitment.
• The formation of an osteo/fibrochondrogenic interface was observed *ex vivo.*

## Graphical abstract

3-L collagen scaffolds that recapitulate the matrix composition of the enthesis are able to promote zonal-specific differentiation of seeded human bone marrow derived mesenchymal stromal cells towards an osteogenic lineage and, with the aid of bioactive molecules, towards a fibrocartilage lineage, in an *in vitro* and *ex vivo* scenario (graphical abstract created with BioRender.com).

**Figure:**
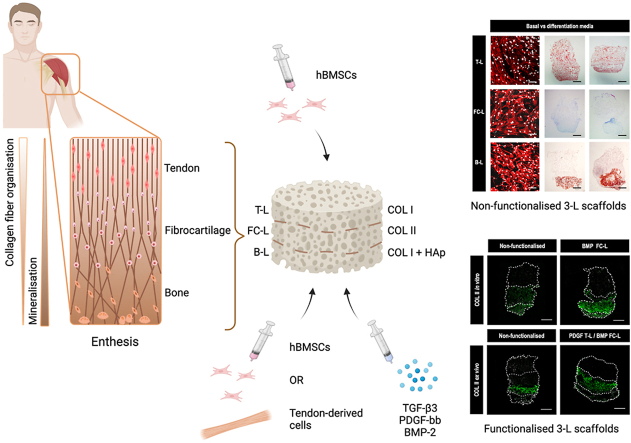

## 1 Introduction

Entheses are specialised insertion sites that connect tendons or ligaments to bones and play a crucial role in the smooth force transition from soft to hard tissue, joint motion and physiological loading transfer [[1], [2], [3], [4]]. The fibrocartilaginous entheses (e.g. rotator cuff, anterior cruciate ligament and Achilles tendon [2]) are typically composed of a fibrous connective tissue (tendon tissue populated by fibroblasts, with a predominant composition of linearly arranged collagen type I fibres); a non-mineralised and a mineralised fibrocartilage region (a mixed composition of collagen types I, II, III and X populated by fibrochondrocytes and hypertrophic chondrocytes); and a bone layer (a matrix composed of collagen type I and hydroxyapatite in which osteoblasts, osteoclasts and osteocytes are embedded in) [5,6]. Injuries, disorders and aging can induce enthesis degeneration [7], which, in the USA alone, is associated with over 300,000 shoulder, foot, and ankle surgical repairs [8] and around 350,000 anterior cruciate ligament reconstructive surgeries [9] and in excess of US$ 30 billion healthcare expenditure [10]. Traditional intervention strategies, such as the direct suturing of bone to tendon or ligament [[11], [12], [13]], largely underestimate the function of this multiphasic tissue, ultimately resulting in poor patient therapeutic outcomes [[14], [15], [16], [17]]. Indeed, failure rates between 20% and 94% have been reported for chronic rotator cuff injuries alone and have been primarily attributed to a poor graft-to-bone integration, which results in a fibrovascular scar instead of the typical multiphasic enthesis structure and a mechanical mismatch with the native tissue [18,19]. Thus, re-establishment of a continuous interface following injury is critical for the long-term success of grafted implants. Towards this goal, the use of decellularised tissues has been advocated [[20], [21], [22]]. Unfortunately, the high density and low porosity of the enthesis tissue prohibit both effective decellularised (without significantly compromising the multi-layered structure of the graft) and repopulation from endogenous cells [23,24]. Other approaches under investigation involve the development of multiphasic scaffolds seeded with adult differentiated cell types [[25], [26], [27], [28]] or adult mesenchymal stromal cells [[29], [30], [31]]. The former strategy exploits the heterotypic interactions between different cell phenotypes (commonly tendon fibroblasts, chondrocytes and osteocytes) in proximity to each other, which, although may result in the formation of an enthesis-like tissue, poses considerable scalability and regulatory challenges [32]. The latter approach benefits from the use of a single cell population (making its applicability far easier), that will differentiate towards the appropriate tissue lineage *in situ* through scaffold- and local microenvironment-induced signals [33]. Recent advances in biomaterial engineering have inspired the development of multi-cargo delivery vehicles [[34], [35], [36]] but, despite the very promising preclinical data of such elegant systems, none of them has been clinically translated yet. This limited technology transfer can be attributed either to the complex composition (e.g. collagens from different species [37] or a combination of different types of materials, like silk fibroin, tri-calcium phosphate and polyether ether ketone anchor [38]) or the intricated fabrication method (e.g. multiple freeze drying steps [39] or a an array of techniques, like the formation of beads, followed by a layer-by-layer coating technique and CO_2_ laser technology [40]) of the device that again jeopardise regulatory clearance and substantially increase costs of goods [[41], [42], [43], [44]]. Therefore, the quest for a scalable, yet multifunctional device for multiphasic insertion sites continuous.

Herein we developed a three-layer (3-L) porcine only collagen-based scaffold (collagen type I, collagen type II, collagen type I and hydroxyapatite) with an one-step cross-linking and freeze drying fabrication method. To further enhance the biological effect, zonal specific bioactive molecules (BMs) [tenogenic differentiation: platelet-derived growth factor bb (PDGF-bb) and transforming growth factor β3 (TGF-β3); fibrochondrogenic differentiation: TGF-β3 and bone morphogenetic protein 2 (BMP-2)] were incorporated in the scaffold during the fabrication process. To validate the potential of the device to induce a layer-specific cell response, human bone marrow mesenchymal stromal cells (hBMSCs) were seeded on the scaffold and their differentiation towards tenogenic, fibrochondrogenic and osteogenic lineages was investigated. Finally, the functionalised 3-L scaffolds were tested in an *ex vivo* model to assess proof of principle.

## 2 Materials and methods

### 2.1 Materials

Porcine Achilles tendons and kneecaps were obtained from a local slaughterhouse. Hydroxyapatite nanopowder (HAp, MW 502.31 ​Da, <200 ​nm size particles) was purchased from Sigma Aldrich (Ireland). 4-arm polyethylene glycol succinimidyl glutarate 10,000 ​Da (PEG-SG) was purchased from JenKem Technology (USA). hBMSCs were purchased from RoosterBio Inc (USA). All tissue culture plasticware were purchased from Sarstedt (Ireland). All chemicals, cell culture media and reagents were purchased from Sigma Aldrich (Ireland), unless otherwise stated.

### 2.2 Collagen type I and II extraction and characterisation

Collagen type I (COL I) [[45], [46], [47]] and collagen type II (COL II) [48,49] were extracted from porcine Achilles tendons and knee caps, respectively, by acid-pepsin digestion and repeated salt-precipitation and centrifugation. Collagen purity was assessed via gel electrophoresis [50] and correlated to purified COL I (Symatese, France) and COL II (Sigma Aldrich, Ireland). Briefly, the extracted and purified collagens were neutralised with sodium hydroxide (NaOH), denatured at 95 °C and resolved under non-reducing conditions using an in-house 5% resolving and a 3% stacking polyacrylamide gels on a Mini-Protean 3 (Bio- Rad Laboratories, UK) system. The gels were stained with a SilverQuest™ Silver Staining Kit (Invitrogen, Ireland) according to the manufacturer's instructions. A Hitachi scanning electron microscope (SEM) S-4700 equipped with an energy dispersive X-ray analysis system (EDX, Hitachi-Hisco Europe GmbH, Germany) operating at a voltage of 10–15 ​keV and magnifications higher than 300x, was employed for assessment of collagen chemical composition. Samples were considered pure when major elements, such as hydrogen, carbon and oxygen, were found and the amounts of sodium and chloride were negligible (<1%). Overall, the in-house extracted COL I and COL II exhibited the typical electrophoretic mobility, comparable to the commercially available preparations (Fig. S1a) and no salt or other contaminants were present (Fig. S1b).

### 2.3 COL I and HAp suspension preparation and characterisation

COL I and HAp suspension (COL I/HAp) was prepared as previously reported [51]. Briefly, COL I was blended with 0.5 ​M acetic acid solution for 90 ​min in a cooled reaction vessel using an overhead blender (IKA Ultra Turrax T18, Works Inc., USA) at a speed of 15,000 ​rpm. HAp was suspended in 0.5 ​M acetic acid solution and added in aliquots to the COL I every 15 ​min for the total duration of the process (90 ​min) at 4 °C to obtain a suspension with a final ratio of 1 part Col I to 1 part HAp (w/w). The solution was left to degas at 4 °C overnight. EDX analysis was employed to obtain a map of the relative distribution of calcium and phosphorous in the suspension. Fourier transform infrared spectroscopy (FT-IR, Varian 660-IR, Ireland) was used to examine the chemical structure of COL I/HAp suspension. Spectra of COL I, HAp and COL I/HAp were collected in the wavelength range of 4000-400 ​cm^−1^ using a spectrometer equipped with diamond crystal (Varian 610-IR, JVA analytical, Ireland). EDX, elemental mapping and FT-IR (Fig. S1c) analyses confirmed the presence of calcium and phosphorus in the COL I/HAp suspension.

### 2.4 3-L collagen scaffold preparation

To fabricate the 3-L collagen scaffolds, the bone-like layer (B-L) was firstly produced by cross-linking the COL I/HAp suspension with 1 ​mM PEG-SG (dissolved in 0.5 ​mM acetic acid) to produce a final solution of 4.5 ​mg/ml. The solution was left at room temperature for 3 ​h to allow cross-linking of collagen. Afterwards, the solution was poured in a silicon mould and frozen at -20 °C. Next, the fibrocartilage-like layer (FC-L) was prepared by cross-linking the solution of COL II with 1 ​mM PEG-SG to a final concentration of 5 ​mg/ml. The suspension was allowed to cross-link at room temperature, poured on top of the already frozen B-L and was let to freeze at -20 °C. The same process was repeated for the tendon-like layer (T-L) by cross-linking (1 ​mM PEG-SG dissolved in 0.5 ​mM acetic acid to a final collagen concentration of 5 ​mg/ml) a COL I solution, which was poured on top of the already frozen FC-L, to obtain the final 3-L scaffold. The frozen construct was subsequently transferred to a stainless-steel mould and freeze dried (VIRTIS® Advantage EL Freeze Dryer, USA) for 1 day, to ensure complete dryness. Cylindrical samples of 6 ​mm diameter and 9 ​mm height were cut from the scaffold using a biopsy punch prior to further analysis. A cross-linked COL I, COL II or COL I/HAp solutions were also individually frozen and freeze dried to produce T-L, FC-L and B-L monolayer scaffolds, respectively.

### 2.5 Physicochemical, resistance to enzymatic degradation, mechanical and morphological analyses

Residual free amines [50], resistance to enzymatic degradation [52] and compression properties [48] were assessed for PEG-SG cross-linked T-L, FC-L and B-L monolayer scaffolds and for 3-L cross-linked and non-cross-linked scaffolds. Residual free amines were analysed with the trinitrobenzenesulfonic acid assay (TNBSA, ThermoFisher Scientific, UK); samples were incubated in 0.1 ​M sodium bicarbonate; 0.01% of TNBSA was added to the samples and incubated for 2 ​h at 37 ​°C. The reaction was stopped and absorbance of each sample was read at 335 ​nm. The free amine groups quantified by interpolating values from a linear standard curve of known concentrations of glycine. Resistance to enzymatic degradation was quantified by weighing approximately 5 ​mg of collagen scaffolds and incubating them in bacterial collagenase type II (MMP-8, 10 U/ml, ThermoFisher Scientific, UK). After 6, 12 and 24 ​h of incubation at 37 ​°C, samples were centrifuged, the supernatant was removed, the remaining pellet was freeze dried and weighed. Enzymatic degradation was quantified as weight loss respect to the initial weight. Mechanical properties were measure by compressive test using a Z009 Zwick Tensile Tester (Zwick Roell, Ireland). Samples were compressed with a deformation rate of 8 ​mm/min and 100% deformation. Stress-strain curves (displayed as the closest curve to the middle value between 3 tested specimens) and ultimate compressive strength were extrapolated from the obtained data.

The structure of the 3-L scaffolds was characterised by macroscopic and micro computed-tomography (μCT) analyses. For μCT analysis, 2 independent samples were scanned at 40 ​kV with a voxel size of 9 ​mm by a Scanco μCT100 system (SCANCO Medical, Switzerland) and characterised using the MicroCT FTP Software, version 3.7 (SCANCO Medical, Switzerland). The pore size of the 3-L scaffolds was characterised by scanning electron microscopy (SEM). For SEM analysis, scaffolds were imaged using a Hitachi S-4700 system at 5.0 ​kV after gold-coating (Emitech K-550X Sputter Coater, Emitech, UK). Quantification of pore size and aspect ratio (minor axis/major axis) was performed with ImageJ software (NIH, USA), by analysing 5 fields of view (FOV) from 3 different scaffolds.

### 2.6 Cell expansion and seeding

hBMSCs from 3 healthy male donors under 30 years old were purchased from RoosterBio Inc (USA) at passage 2 and expanded in RoosterNourish™ medium following the manufacturer's protocol. Prior to cell seeding, the scaffolds were sterilised in 70% ethanol solution for 30 ​min and rinsed 3 times with 1x phosphate buffered saline (PBS). hBMSCs at passages 4 were harvested from culture flasks using trypsin-ethylenediaminetetraacetic acid. Cells were washed with PBS and centrifuged at 800 ​g for 5 ​min. The cell pellet was resuspended in basal medium [consisting of α-minimal essential medium (αMEM GlutaMax™, ThermoFisher Scientific, UK) supplemented with 10% foetal bovine serum (FBS) and 1% penicillin/streptomycin (PS)]. The cell suspension was seeded onto the scaffolds at a density of 450,000 ​cells per scaffold. To ensure complete penetration of the cells into the scaffold, a syringe vacuum-assisted method was used [53]. Briefly, the scaffolds were placed in a 5 ​ml syringe and the cell suspension was aspired. The entrance of the syringe was closed by using a luer lock cap and the plunger was pulled back to create vacuum, held for 10 ​s and positioned back to the initial position for 10 more sec. These steps were repeated 3 times before placing the scaffolds in the well plate and in the incubator. Medium was refreshed every 3 days for the duration of all experiments.

### 2.7 Cell viability, metabolic activity, proliferation and morphometric analyses

Cell viability, metabolic activity and proliferation were assessed after 3, 7 and 21 days in culture. Cell viability and metabolic activity were analysed employing LIVE/DEAD® and alamarBlue® assays (ThermoFisher Scientific, Ireland), respectively, as per manufacturer's protocols. Cell metabolic activity was expressed as percentage reduction of the alamarBlue® dye and normalised to DNA content. This was measured after scaffold digestion in a solution of 50 ​μg/ml proteinase K in dipotassium phosphate solution at pH 8.0 and 56 °C overnight and subsequent incubation of the supernatant with Quant-iT™ PicoGreen® reagent (Quant-iT™ PicoGreen® dsDNA assay, Life Technologies, Ireland). To assess cell morphology, scaffolds were fixed with 4% paraformaldehyde, permeabilised with 0.2% Triton X-100 and the cytoskeleton was stained with rhodamine labelled phalloidin (Life Technologies, Ireland) and the nuclei with 4′,6-diamidino-2-phenylindole (DAPI, ThermoFisher Scientific, Ireland). Fluorescent images for cell viability and morphometric analyses were captured using an OlympusFluoview1000 Shackleton confocal microscope (Olympus, Ireland). Quantification of cell and nuclear area in each of the 3-L was performed using ImageJ software. Cell alignment was calculated through manual measurement of individual cell cytoskeleton angle to quantify the distribution of aligned cells respect to the pore direction in the T-L, with ImageJ software. A minimum of 5 FOV from 3 biological replicates were imaged for morphometric analysis.

### 2.8 hBMSC culture in tenogenic, chondrogenic and osteogenic media

For tenogenic lineage commitment, hBMSCs were cultured at passage 3 in MesenCult™ -ACF Plus Medium (StemCell Technologies, UK) supplemented with L-glutamine (1–100), 1% PS and a supplement (StemCell Technologies, UK). Afterwards, cells were trypsinised and seeded on the scaffolds. Cells were allowed to attach and spread for 3 days in MesenCult™-ACF Plus Medium. Tenogenic differentiation was induced with MesenCult™ tenogenic differentiation medium (StemCell Technologies, UK) at day 4.

For chondrogenic lineage commitment, cell seeded scaffolds were cultured in basal medium for 3 days before switching to a chondrogenic differentiation medium, consisting of 100 ​nM dexamethasone, 100x insulin, transferrin, sodium selenite and linoleic-bovine serum albumin supplement (ITS ​+ ​1 liquid media supplement), 40 ​μg/ml L-proline, 50 ​μg/ml ascorbic acid-2-phosphate, 10 ​ng/ml of transforming growth factor β3 (TGF-β3) in Dulbecco's Modified Eagle Medium High-Glucose (DMEM, ThermoFisher Scientific, Ireland) supplemented with 1% PS.

For osteogenic lineage commitment, cell seeded scaffolds were cultured in basal medium for 3 days before switching to an osteogenic differentiation medium, composed of 10 ​mM β-glycerophosphate disodium salt hydrate, 100 ​nM dexamethasone, 50 ​μM ascorbic acid-2-phosphate in basal medium.

For all cell culture experiments, fresh medium was replaced every 3 days for the whole duration of the incubation time (up to day 21).

### 2.9 3-L scaffold characterisation in basal and differentiation media

Cell seeded scaffolds were rinsed in PBS and fixed with 4% paraformaldehyde overnight at day 7 and day 21. Afterwards, paraffin-embedded slides were obtained with a tissue processor (ThermoFisher Scientific Excelsior™ ES Tissue Processor, UK). 10 ​μm thick sections were cut using a microtome (Leica Biosystems, UK), dried at 60 °C for 1 ​h and stored at room temperature for further histological characterisation.

Tenogenic differentiation was assessed after 7 and 21 days by picrosirius red staining (Direct Red 80, Sigma Aldrich, Ireland) on scaffolds cultured in basal and tenogenic medium, according to the manufacturer's guidelines. Quantification of stained area was performed by Image J. Stained area of acellular scaffolds (Fig. S2a) was subtracted from the experimental values.

Chondrogenic differentiation was assessed after 7 and 21 days by Alcian blue (8 GX, Sigma Aldrich, Ireland) and nuclear fast red (Sigma Aldrich, Ireland) staining on scaffolds cultured in basal and chondrogenic medium, according to the manufacturer's guidelines. Quantification of Alcian blue stained area was performed by ImageJ. Complementary quantification of glycosaminoglycans (GAGs) was performed with the Glycosaminoglycan assay Blyscan™ kit (Biocolor, UK) as per manufacturer's protocol [54], after dissociation of the three layers with the aid of a stereoscope and scalpel. Briefly, samples were digested with proteinase K and the supernatant was collected. A standard curve was generated using bovine tracheal chondroitin 4-sulphate standard. Supernatants from samples and standards were incubated with the dye reagent. After agitation, the samples were centrifuged and the supernatants were discarded without disrupting the pellet. A dye dissociator was added to the samples and mixed. Absorbance was measured at 656 ​nm using a Varioskan Flash spectral scanning multimode reader (ThermoFisher Scientific, UK). The GAG content of the pellets was normalised to the amount of DNA.

Osteogenic differentiation was assessed after 7 and 21 days by alizarin red staining (Sigma Aldrich, Ireland) on scaffolds cultured in basal and osteogenic medium, according to the manufacturer's guidelines. Quantification of stained area was performed by ImageJ. Stained area of acellular scaffolds (Fig. S2b) was subtracted from the experimental values. For calcium quantification, a StanBio Calcium Liquicolour™ Kit (ThermoFisher Scientific, Ireland) was used, as per manufacturer's protocol. Absorbance at 550 ​nm was measured using the Varioskan Flash spectral scanning multimode reader and the amount of calcium per well was calculated using calcium standards and normalised by the amount of DNA. To assess alkaline phosphatase (ALP) activity, scaffolds were digested in proteinase K solution and the supernatant was incubated with 1-Step™ PNPP Substrate Solution (ThermoFisher Scientific, UK). Absorbance at 405 ​nm was measured using a Varioskan Flash spectral scanning multimode reader. Amount of p-nitrophenol was calculated using p-nitrophenol standards and units of enzyme were calculated dividing the μmoles of p-nitrophenol produced by the time. Units of enzyme were normalised by the amount of DNA. Both calcium quantification and ALP activity analysis were performed on 3-L scaffolds cultured as a whole in basal and osteogenic media and subsequently dissociated in the three layers.

Gene expression analysis was conducted on 3-L scaffolds cultured as a whole in basal and differentiation media and subsequently separated in the three layers. hBMSCs cultured on tissue culture plastic (TCP, same cell density and culture conditions of the scaffolds) were used as a control and RNA extracted from these cells was used for normalisation. RNA extraction was performed as previously described [55] by recovering the RNA phase with chloroform and eluting it through High Pure RNA isolation columns (Roche, Ireland). Total RNA concentration and quality were analysed using the NanoDrop 1000 (ThermoFisher Scientific, Ireland) and the Agilent 2100 Bioanalyser (Agilent Technologies, Ireland). RNA was transcribed to cDNA using a Transcriptor First Strand cDNA synthesis kit (Roche, Ireland). The expression of tenogenic [collagen type I (COL1A1) and III (COL3A1), scleraxis (SCXA), tenomodulin (TNMD), tenascin-C (TNC), mohawk (MKX)], chondrogenic [collagen types II (COL2A1) and X (COL10A1), sex-determining region Y-box 9 (SOX9)] and osteogenic [runt-related transcription factor 2 (RUNX2), bone gamma-carboxyglutamate protein (BGLAP), secreted phosphoprotein 1 (SPP1)] markers was assessed by qPCR, which was performed with a StepOnePlus™ Real-Time PCR System (ThermoFisher Scientific, Ireland), using TaqMan primer probe assays (IDT, Belgium, listed in Table S1) and TaqMan Gene Expression Mastermix (ThermoFisher Scientific, Ireland). The amplification conditions were 50 ​°C for 2 ​min, 95 ​°C for 10 ​min, followed by 40 cycles of 95 ​°C for 15 ​s and 60 ​°C for 1 ​min. The analysis was performed at day 7 and 21, as it was not possible to gather sufficient RNA at day 3; 3 biological replicates were analysed by pulling together 6 technical replicates per time point. Values of targeted genes were normalised to three reference genes [glyceraldehyde 3-phosphate dehydrogenase, 60S acidic ribosomal protein P0 and β2 microglobulin] (ΔCt) and to cells cultured on TCP (ΔΔCt). Z-scores of fold changes were calculated and relevant up- and down-regulations were accepted when score was at least two standard deviations away from the mean value of fold-change for each gene.

### 2.10 Screening of BMs supplemented in basal medium

Platelet-derived growth factor-bb (PDGF-bb), TGF-β3, insulin-like growth factor 1 (IGF-1), growth differentiation factors 7 and 5 (GDF-7 and GDF-5) were tested for tenogenic differentiation of hBMSCs on T-L monolayer scaffolds. Bone morphogenetic protein 2 (BMP-2), TGF-β3, IGF-1, hyaluronic acid and kartogenin were tested for chondrogenic differentiation of hBMSCs on FC-L monolayer scaffolds (Table S2 for suppliers, concentration used and rational of selection). Cell density was adapted to be 1/3 of the initial amount required for the 3-L scaffolds and all the molecules were supplemented in basal medium. The medium was changed every 3 days up to 21 days. Afterwards, the scaffolds were processed to obtain paraffin-embedded slices. After blocking for 1 ​h at room temperature in 5% PBS/bovine serum albumin (BSA), the sections were incubated with primary antibodies diluted in blocking buffer for 3 ​h, followed by 3 washes in PBS. To assess tenogenic differentiation, COL I (BosterBio, USA), collagen type III (COL III, Abcam, UK) and tenascin (TNC, Abcam, UK) were used; for chondrogenic differentiation, COL II (Abcam, UK), collagen type X (COL X, Abcam, UK) and chondroitin sulphate (CS, Sigma Aldrich, Ireland) were used (Table S3 provides antibody details). Subsequently, secondary antibody solutions were added for 1 ​h at room temperature, followed by 3 washes in PBS. Nuclei were stained with DAPI and sections were mounted with Fluoromount™ Aqueous Mounting Medium (Sigma-Aldrich, Ireland). Images were taken with a FV3000 Fluoview Confocal Laser Scanning Biological Microscope (Olympus, Ireland). Quantification of stained area was carried out in ImageJ by quantifying 3 FOV per scaffold from 3 biological replicates.

### 2.11 Functionalisation of the T-L and FC-L of the 3-L scaffolds with BMs

PDGF-bb and TGF-β3 were chosen to functionalise the T-L and BMP-2 and TGF-β3 were chosen to functionalise the FC-L of the 3-L scaffolds. Either a single layer functionalisation (i.e. single functional molecule in the 3-L scaffold) or a double layer functionalisation (i.e. one functional molecule per layer in the 3-L scaffold) was performed. For the single layer functionalisation, the T-L solution was prepared to obtain a final concentration of 0.1 ​μg/ml PDGF-bb or TGF-β3 and was left to cross-link at room temperature for 3 ​h (from now on called PDGF in T-L and TGF in T-L scaffolds). The functionalised T-L was then poured on top of the already frozen FC-L and B-L and freeze dried. Similarly, a FC-L solution was prepared to obtain a final concentration of 0.05 ​μg/ml of TGF-β3 or 0.3 ​μg/ml BMP-2 and was left to cross-link at room temperature for 3 ​h (from now on called TGF in FC-L and BMP in FC-L). The functionalised FC-L was then poured on top of the already frozen B-L and the day after the non-functionalised T-L was layered on top of it. The final construct was then freeze dried. For the double layer functionalisation, four combinations of the selected molecules were tested (from now on called PDGF in T-L/TGF in FC-L; PDGF in T-L/BMP in FC-L; TGF in T-L/TGF in FC-L; TGF in T-L/BMP in FC-L). Scaffolds were seeded with hBMSCs and cultured in basal medium. Half of the medium was changed every 3 days with fresh basal medium up to 21 days.

### 2.12 Characterisation of the functionalised 3-L scaffolds

To assess cell morphology and cell penetration into the functionalised and non-functionalised scaffolds, samples were stained with rhodamine labelled phalloidin after 21 days. At day 7 and 21, samples were also stained for COL I and TNC and with COL II and COL X to assess hBMSC tenogenic and chondrogenic differentiation, respectively. Images were taken with a FV3000 confocal microscope and the full length of the scaffolds was reconstructed by multi-area time lapse (MATL); stained area was measured by quantifying 3 FOV per scaffold from 3 biological replicates with ImageJ.

### 2.13 *Ex vivo* assessment of the 3-L scaffolds

3 adult female rats were euthanised for reasons unrelated to this research in accordance with the European (EU) guidelines (2010/63/UE), Health Products Regulatory Authority of Ireland and the Animal Care Research Ethics Committee of the National University of Ireland, Galway. Every effort was made to minimise animal suffering, pain or distress and to reduce the number of animals used, for example by sharing tissue with other researchers. Achilles tendons of the posterior paws were isolated, washed in cold PBS, plated in 6 well plate and let attach for 2 ​h. After this, the non-functionalised and the double functionalised 3-L scaffolds were placed in the well plates, with the T-L in proximity of the extremity of the tendons and 600 ​μl of basal medium were added to cover the tissue and the scaffold. The medium was changed every 3 days for 21 days (sufficient time to allow cells to migrate outside the tendons). At this point, the tendon tissue was removed and the scaffolds were kept in culture for 21 more days. The samples were then collected, rinsed, fixed and stained with rhodamine labelled phalloidin. Scaffolds were also paraffin-embedded for histological observations (picrosirius red, Alcian blue and alizarin red stainings) and immunohistochemistry (COL I and COL II stainings). Quantification of COL I and II stained area was performed with ImageJ.

### 2.14 Statistical analysis

A minimum of three independent experiments were performed, unless otherwise mentioned, and data depicted as mean ​± ​standard deviation (SD). Data were analysed using the GraphPad v6.01 (GraphPad Software Inc., USA) software. Student's t-test and one-way or two-way ANOVA followed by Fisher's post-hoc test were employed after confirming normal distribution for each sample population (Kolmogorov-Smirnov normality test) and equality of variances (Levine's test for homogeneity of variance). When these conditions were not met, Mann-Whitney *U* test and Kruskal-Wallis test were employed to assess significant differences. Results were considered significant for p ​< ​0.05.

## 3 Results

### 3.1 3-L scaffold morphological and physicochemical characterisation

**Fig. 1:**
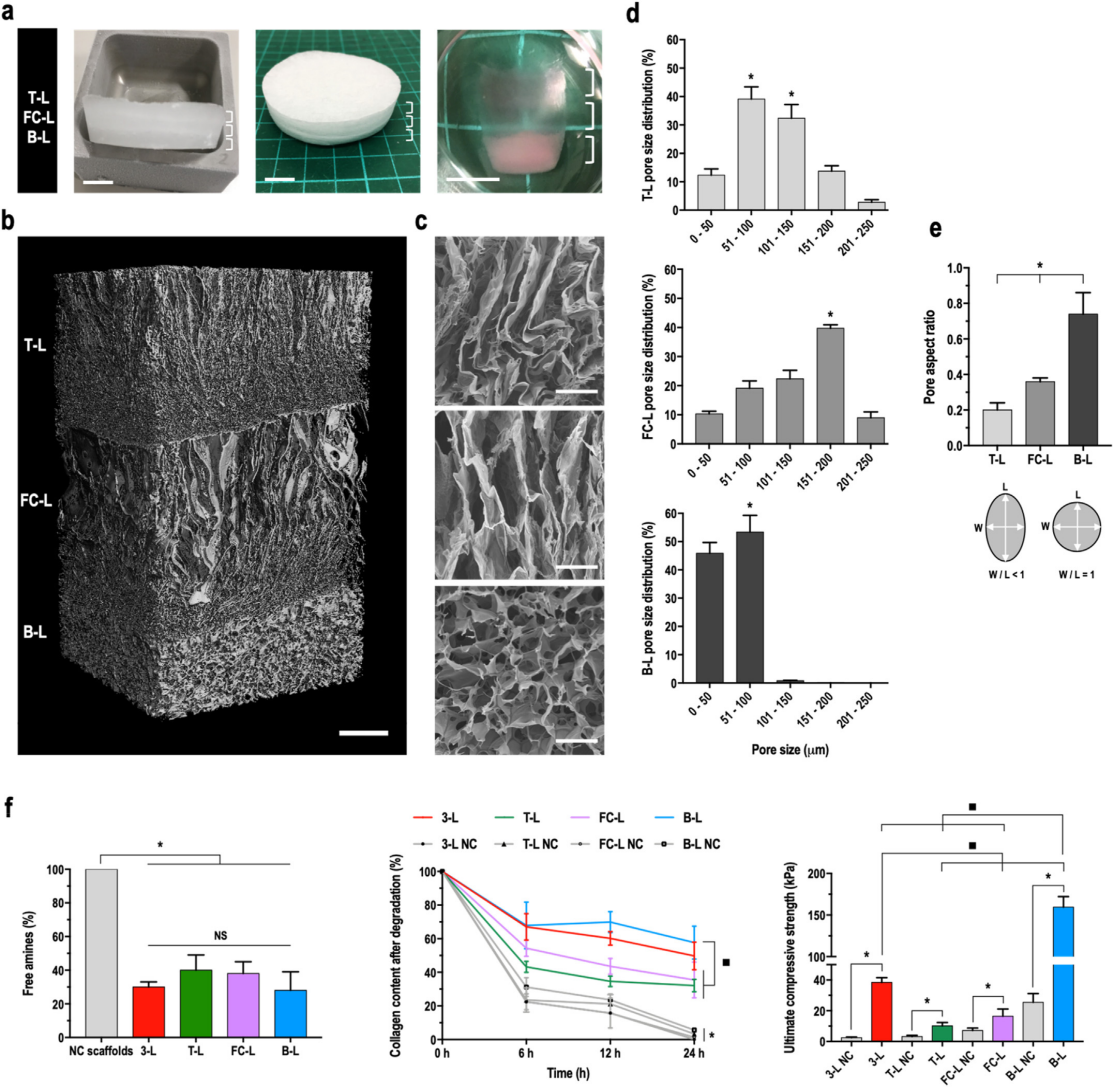
Cross-linked 3-L scaffolds present three distinct yet integrated layers with zone-specific pore structures. **a**) Macroscopic observation of 3-L scaffolds before freeze drying, after freeze drying and in basal medium (pink colour due to phenol red), showing the presence of three layers; scale bar 1.5 ​cm. **b**) μCT analysis, showing a longitudinal section of the scaffold; scale bar 1 ​mm. **c**) SEM analysis of the three layers showing elongated pores in the T-L and FC-L and round pores in the B-L; scale bar 50 ​μm. **d**) Distribution (%) of pore size (μm) across the three layers **e**) Pore aspect ratio (W ​= ​width and L ​= ​length). **f)** Free amine content (%) measured with TNBSA assay; **g**) Collagen content after degradation (%) in collagenase type II. **h**) Ultimate compressive strength values (kPa) after compression tests. The three assays were performed on non-cross-linked (NC, in grey) 3-L scaffolds, T-L, FC-L and B-L monolayer scaffolds and on PEG cross-linked 3-L scaffolds (red), T-L (green), FC-L (purple) and B-L (blue) monolayer scaffolds. All data presented as mean ​± ​SD and N ​= ​3, except μCT analysis (N ​= ​2). ∗ indicates significant (p ​< ​0.05) difference between pore size distribution values (**d**), layers (**e**) or cross-linked vs non cross-linked scaffolds (**f**, **g** and **h**); ▪□ indicates significant (p ​< ​0.05) difference between PEG cross-linked 3-L scaffolds and monolayer scaffolds. (For interpretation of the references to colour in this figure legend, the reader is referred to the Web version of this article.)

Macroscopic (Fig. 1a) and μCT (Fig. 1b) analyses of the PEG-SG cross-linked 3-L scaffolds confirmed that the scaffolds were comprised of three interconnected, yet distinguishable layers. SEM (Fig. 1c) and complementary pore size (Fig. 1d) and aspect ratio (Fig. 1e) analyses made apparent that the T-L and the FC-L presented a large and elongated pore structure and the B-L presented a small and round pore structure. PEG-SG cross-linking of the 3-L scaffolds and the T-L, FC-L and B-L monolayer scaffolds resulted in significantly (p ​< ​0.001) lower free amine content (Fig. 1f), significantly (p ​< ​0.001) higher resistance to collagenase type II digestion (Fig. 1g) and significantly (p ​< ​0.001) higher ultimate compressive strength (Fig. 1h) than the non-cross-linked 3-L collagen scaffolds and the T-L, FC-L and B-L monolayer scaffolds, respectively. Non-cross-linked and cross-linked B-L monolayer scaffolds exhibited significantly (p ​< ​0.05) higher resistance to enzymatic degradation (Fig. 1g) and ultimate compressive strength (Fig. 1h) than the respective non-cross-linked and cross-linked T-L and FC-L monolayer scaffolds, possibly due to the presence of HAp in the B-L.

### 3.2 Cytocompatibility and cell morphometric analyses of hBMSCs seeded on non-functionalised 3-L scaffolds

**Fig. 2:**
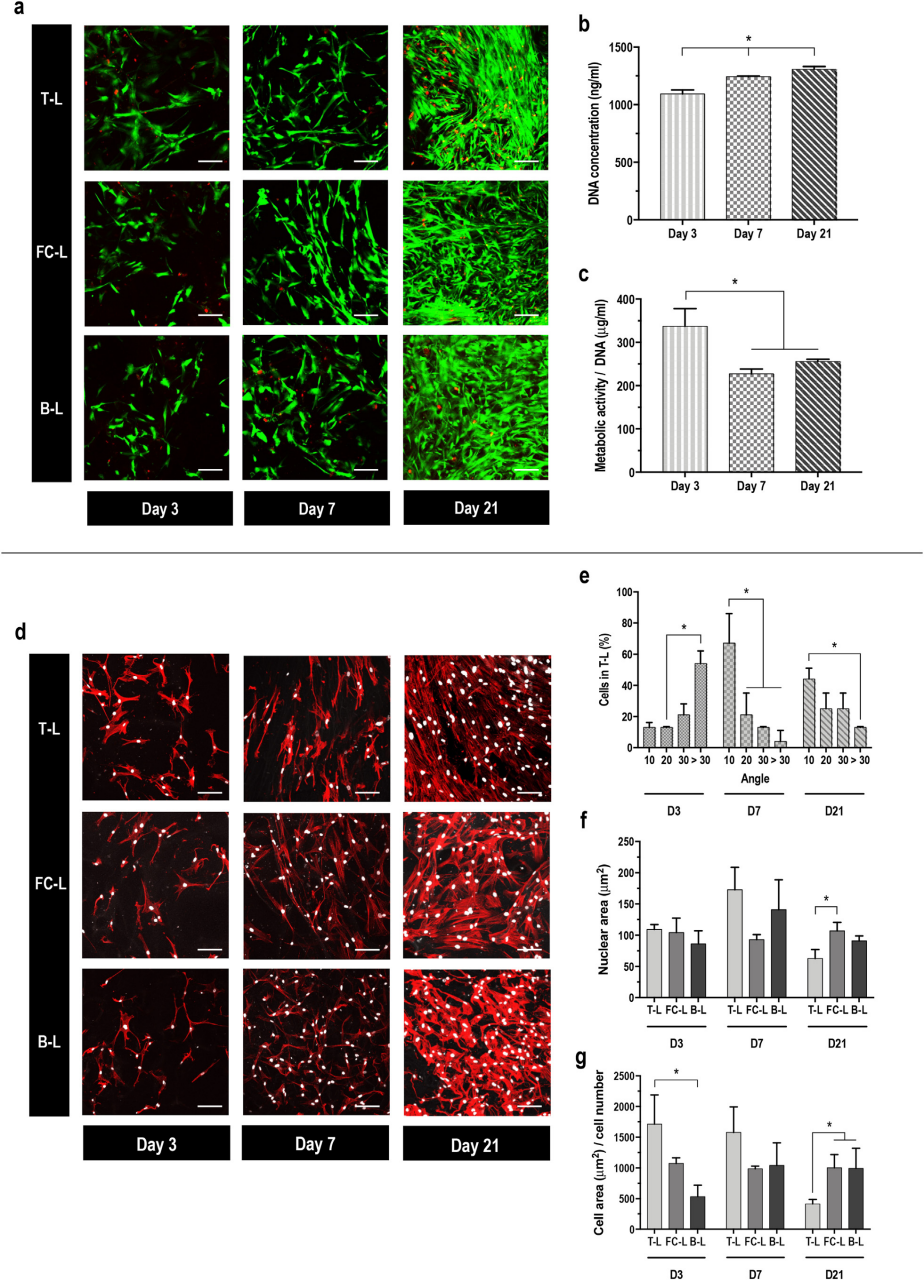
hBMSCs seeded onto 3-L non-functionalised scaffolds spread homogeneously and present a bidirectional cell orientation. **a**) Cell viability staining (alive in green, dead in red) of hBMSCs cultured on 3-L scaffolds in basal medium at day 3, 7 and 21; scale bar 100 ​μm. **b**) DNA concentration (ng/ml) measured by Picogreen assay. **c**) Metabolic activity normalised by DNA (μg/ml) measured by alamarBlue™ at day 3, 7 and 21. **d**) Cytoskeleton of cells in the T-L, FC-L and B-L stained with rhodamine labelled phalloidin (red) and nuclei with DAPI (white) after 3, 7 and 21 days in culture; scale bar 100 ​μm. **e**) Percentage of aligned cells in the T-L, at day 3, 7 and 21. **f**) Nuclear area (μm^2^) of cells in the T-L, FC-L and B-L, at day 3, 7 and 21. **g**) Cell area (μm^2^) normalised by cell number of cells in the T-L, FC-L and B-L, at day 3, 7 and 21. All data presented as mean ​± ​SD and N ​= ​3. ∗ indicates significant (p ​< ​0.05) difference between days of culture (**b** and **c**), angle of alignment (**e**) or layers (**f** and **g**). (For interpretation of the references to colour in this figure legend, the reader is referred to the Web version of this article.)

Qualitative viability (Fig. 2a) analysis revealed that all layers equally supported hBMSC growth at a given time point and that the cell number was increased as a function of time in culture. Quantitative DNA concentration (Fig. 2b) analysis revealed a significant (p ​< ​0.05) increase as a function of the time in culture and metabolic activity (Fig. 2c) analysis showed a significant (p ​< ​0.005) decrease from day 3 to day 7 and day 21.

Qualitative assessment of cell morphology (Fig. 2d) revealed a homogeneous spreading of hBMSCs throughout the scaffold and a certain degree of cell alignment in the T-L, starting after 7 days of culture, which was confirmed by quantification of aligned cells (Fig. 2e). By day 21, cells in the T-L were found to have a smaller (p ​< ​0.01) nuclear area in comparison to cells in the FC-L (Fig. 2f) and a smaller (p ​< ​0.05) cell area in comparison to cells in the FC-L and B-L (Fig. 2g).

### 3.3 Characterisation of hBMSC-seeded 3-L scaffolds in basal and differentiation media

**Fig. 3:**
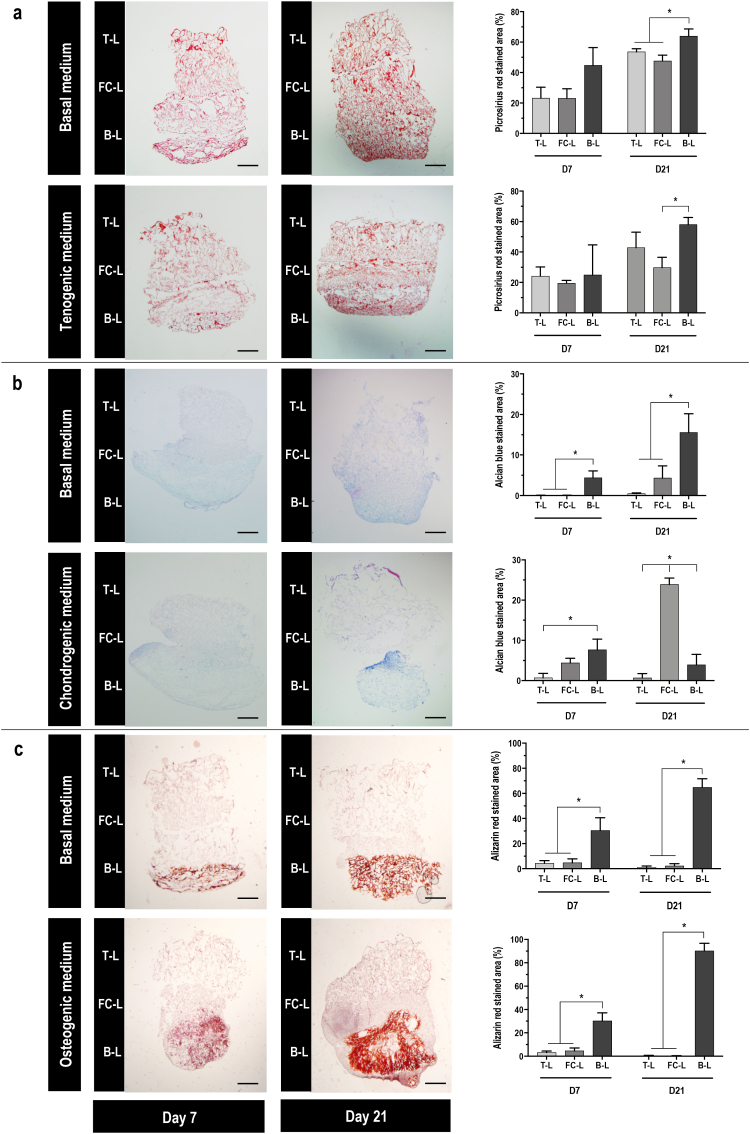
3-L non-functionalised scaffold collagenous composition drives hBMSC differentiation towards tenogenic, chondrogenic and osteogenic lineages. **a**) On the left, picrosirius red staining for collagen total content on paraffin slides of cells cultured on 3-L scaffolds in basal and tenogenic media at day 7 and day 21; scale bar 2 ​mm. On the right, quantification of the staining in the T-L, FC-L and B-L. **b**) On the left, alcian blue and nuclear fast red staining for GAG deposition on paraffin slides of cells cultured on 3-L scaffolds in basal and chondrogenic media at day 7 and day 21; scale bar 2 ​mm. On the right, quantification of the staining in the T-L, FC-L and B-L. **c**) On the left, alizarin red staining for calcium deposition on paraffin slides of cells cultured on 3-L scaffolds in basal and osteogenic media at day 7 and day 21; scale bar 2 ​mm. On the right, quantification of the staining in the T-L, FC-L and B-L. All data presented as mean ​± ​SD and N ​= ​3. ∗ indicates significant (p ​< ​0.05) difference between layers for each time point. (For interpretation of the references to colour in this figure legend, the reader is referred to the Web version of this article.)

Qualitative and quantitative histological analyses for picrosirius red staining (Fig. 3a) of hBMSC-seeded 3-L scaffolds cultured in basal and tenogenic media revealed no significant (p ​> ​0.05) differences in collagen total content between the different layers at day 7. At day 21, instead, the B-L induced the highest (p ​< ​0.005) picrosirius red signal when scaffolds were cultured in basal medium, and a significantly (p ​< ​0.05) higher picrosirius red signal over the FC-L, in tenogenic medium.

Alcian blue staining (Fig. 3b) analysis of hBMSC-seeded 3-L scaffolds cultured in basal and chondrogenic media revealed that when the cells were cultured in basal medium, GAG deposition was significantly (p ​< ​0.05) higher in the B-L in comparison to the other layers at both time points; when the cells were cultured in chondrogenic medium, instead, no significant (p ​> ​0.05) difference was observed between the FC-L and the B-L at day 7 and the FC-L induced the highest (p ​< ​0.001) signal at day 21, suggesting a higher presence of GAGs in this layer. Similarly, quantification of total GAG content (Fig. S3) at both time points demonstrated that when the cells were cultured in basal medium, the B-L induced significantly (p ​< ​0.001) higher total GAG synthesis than the other two layers, and when cells were cultured in chondrogenic medium the FC-L induced significantly (p ​< ​0.001) higher total GAG synthesis than the other two layers.

Alizarin red staining (Fig. 3c) analysis of hBMSC-seeded 3-L scaffolds cultured in basal and osteogenic media at both time points showed a significantly (p ​< ​0.001) higher presence of calcium in the B-L as compared to the other two layers. Likewise, the B-L in both basal and osteogenic media and at both time points induced significantly (p ​< ​0.001) higher ALP activity (Fig. S4a) and calcium deposition (Fig. S4b) than the other two layers.

Genomic (Fig. S5a) analysis of hBMSC-seeded 3-L scaffolds cultured in basal and tenogenic media revealed upregulation (>2 fold change) of COL1A1, COL3A1, TNMD and TNC throughout the 3-L scaffolds and at both time points, in comparison to TCP. At day 7, SCX was upregulated (>2 fold change) exclusively in the T-L in tenogenic medium (unchanged in all other conditions) and at day 21 it was upregulated in all conditions with the exception of B-L in basal medium, where remained unchanged. MKX remained unchanged at day 7 in basal medium in the T-L and at day 21 in the FC-L and B-L and downregulated (<2 fold change) in all other conditions. Genomic (Fig. S5b) analysis of hBMSC-seeded 3-L scaffolds cultured in basal and chondrogenic media revealed upregulation (>2 fold change) of COL10A1 throughout the 3-L scaffolds and at both time points, in comparison to TCP. COL2A1 was only upregulated (>2 fold change) in the FC-L of scaffolds cultured in chondrogenic medium at day 7 and throughout the 3-L scaffolds in chondrogenic medium at day 21 (not detected in all other conditions). SOX9 was upregulated (>2 fold change) throughout the 3-L scaffolds in chondrogenic medium at day 21; it was not detected neither in basal nor in chondrogenic media at day 7 and was unchanged in basal medium at day 21. Genomic (Fig. S5c) analysis of hBMSC-seeded 3-L scaffolds cultured in basal and osteogenic media revealed upregulation (>2 fold change) of RUNX2 in all tested conditions (except in the T-L of scaffolds cultured in basal medium at day 7, where it was unchanged). In general, SPP1 was also upregulated (>2 fold change) in almost all conditions, but the T-L of scaffolds cultured in basal medium at day 7 and throughout the 3-L scaffolds at day 7 in osteogenic medium. BGLAP was only upregulated (>2 fold change) in the B-L in basal medium at both time points; was downregulated (<2 fold change) throughout the 3-L scaffolds at day 21 in osteogenic medium; was not detected at day 7 in the T-L in osteogenic medium; and was unchanged in the remaining conditions.

### 3.4 Characterisation of functionalised 3-L scaffolds for tenogenic differentiation

**Fig. 4:**
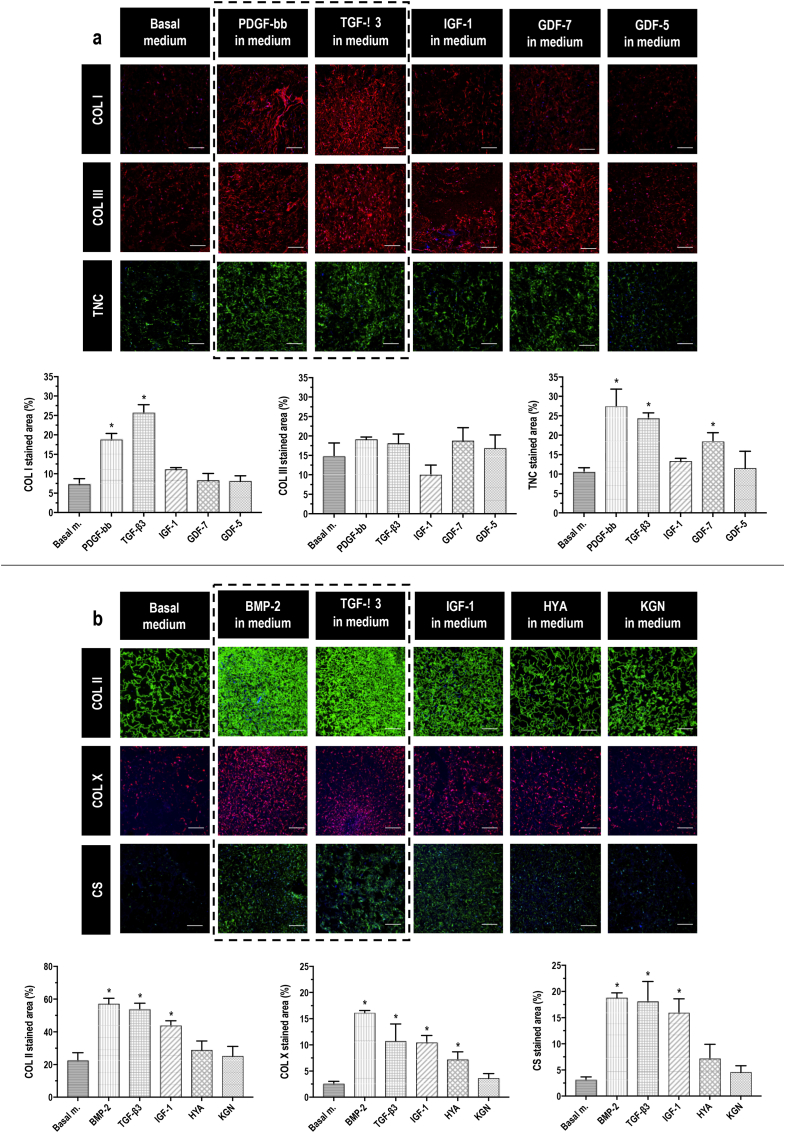
PDGF-bb, TGF-β3 and BMP-2 promote tenogenic and chondrogenic hBMSC differentiation when supplemented in basal medium. **a**) On top, COL I (red), COL III (red), TNC (green) and DAPI (blue) stainings on paraffin slides of cells cultured on T-L monolayer scaffolds in basal medium alone and basal medium supplemented with PDGF-bb, TGF**-**β3, IGF-1, GDF-7 and GDF-5, day 21; scale bar 200 ​μm. On the bottom, quantification of COL I, COL III and TNC stained area (%). **b**) On top, COL II (green), COL X (red), CS (green) and DAPI (blue) stainings on paraffin slides of cells cultured on FC-L monolayer scaffolds in basal medium alone and basal medium supplemented with BMP-2, TGF**-**β3, IGF-1, hyaluronic acid (HYA) and kartogenin (KGN), day 21; scale bar 200 ​μm. On the bottom, quantification of COL II, COL X and CS stained area (%). Within the dashed black boxes, the best molecules. All data presented as mean ​± ​SD and N ​= ​3. ∗ indicates significant (p ​< ​0.05) difference for each molecule compared to basal medium alone. (For interpretation of the references to colour in this figure legend, the reader is referred to the Web version of this article.)

Immunohistochemistry and complementary image intensity analyses (Fig. 4a) at day 21 revealed that, among the BMs assessed in basal medium to induce hBMSC tenogenic commitment on T-L monolayer scaffolds, the PDGF-bb and TGF-β3 significantly (p ​< ​0.001) increased COL I synthesis; no GF affected (p ​> ​0.05) COL III synthesis; and PDGF-bb, TGF-β3 and GDF-7 significantly (p ​< ​0.05) increased TNC synthesis (all in comparison to basal medium alone). Therefore, PDGF-bb and TGF-β3 were chosen as the most promising candidates for the T-L functionalisation.

Qualitative and quantitative assessment of cellular distribution (Fig. S6) within the non-functionalised and the functionalised 3-L scaffolds (all cultured in basal medium) revealed a homogenous cell distribution throughout, at day 21. No statistical difference (p ​> ​0.05) emerged between layers or between conditions.

**Fig. 5:**
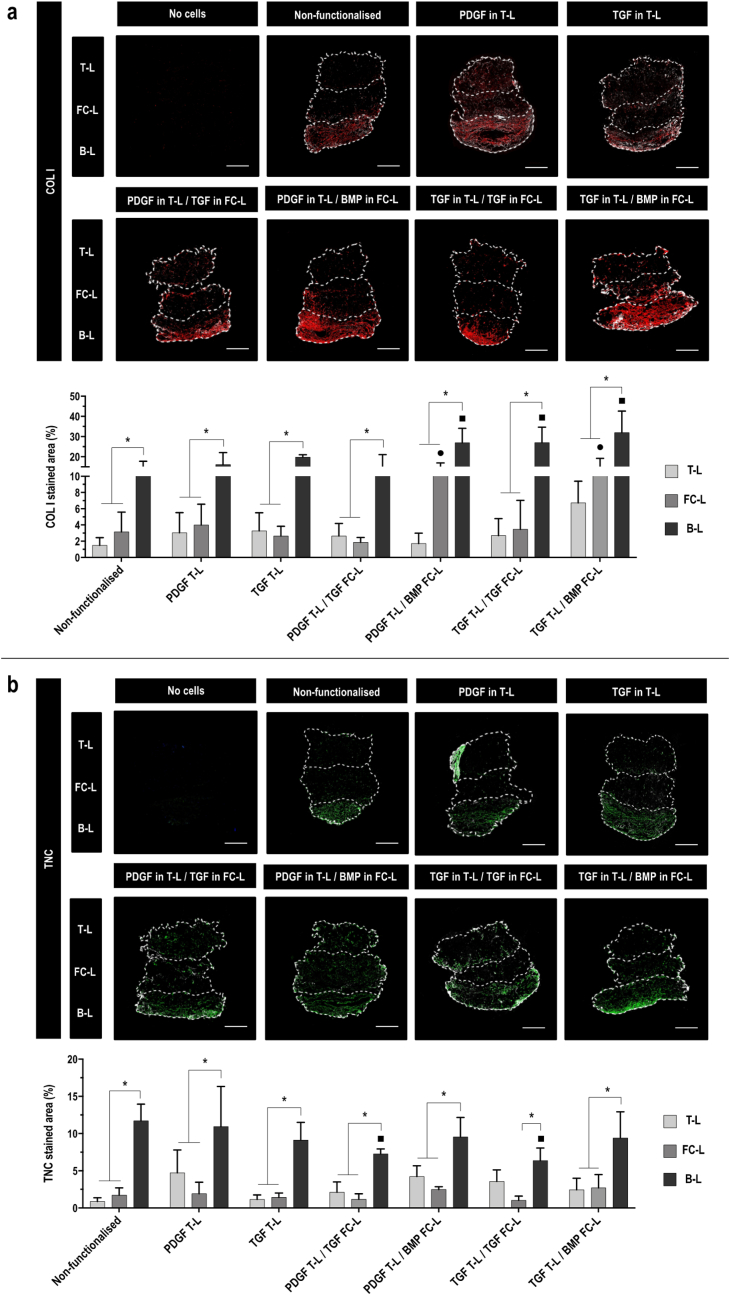
3-L functionalised scaffolds promote a COL I and TNC deposition comparable to non-functionalised scaffolds in the T-L. **a**) COL I (red) and DAPI (white) fluorescent staining on paraffin slides and area quantification (%) at day 21 and **b**) TNC (green) and DAPI (white) fluorescent staining on paraffin slides and area quantification (%) at day 21 of acellular scaffolds (negative control) and hBMSCs cultured on 3-L scaffolds non-functionalised and functionalised with 1 ​GF in the T-L (PDGF in T-L and TGF in T-L) or 2 ​GFs in the T-L and FC-L (PDGF in T-L/TGF in FC-L, PDGF in T-L/BMP in FC-L, TGF in T-L/TGF in FC-L, TGF in T-L/BMP in FC-L); scale bar 500 ​μm. All data presented as mean ​± ​SD and N ​= ​3. ∗ indicates significant (p ​< ​0.05) difference between layers. • indicates significant (p ​< ​0.05) difference between FC-L of functionalised scaffolds vs FC-L of non-functionalised scaffolds. ▪□ indicates significant (p ​< ​0.05) difference between B-L of functionalised scaffolds vs B-L of non-functionalised scaffolds. (For interpretation of the references to colour in this figure legend, the reader is referred to the Web version of this article.)

Immunohistochemistry analysis of COL I at day 7 (Fig. S7a) on non-functionalised and functionalised 3-L scaffolds, revealed a fainted staining in all tested conditions and, overall, no statistical difference (p ​> ​0.05) in COL I deposition emerged between layers or between conditions. Immunohistochemistry analysis of COL I at day 21 (Fig. 5a) revealed a significantly (p ​< ​0.05) higher presence of COL I in the B-L compared to the rest of the scaffold, in all tested conditions. The scaffolds functionalised with PDGF in T-L/BMP in FC-L and TGF in T-L/BMP in FC-L significantly (p ​< ​0.05) promoted COL I synthesis in the FC- L and in the B-L in comparison to the control. The scaffolds functionalised with TGF in T-L/TGF in FC-L significantly (p ​< ​0.05) promoted COL I synthesis in the B-L in comparison to the control. No background staining was observed in acellular 3-L scaffolds from the porcine COL I, which was used to develop the T-L and the B-L.

Immunohistochemistry analysis of TNC at day 7 (Fig. S7b) on non-functionalised and functionalised 3-L scaffolds, made apparent a fainted staining in all tested conditions. Overall, no significant (p ​> ​0.05) difference in TNC deposition was detected between layers or conditions. Immunohistochemistry analysis of TNC at day 21 (Fig. 5b) revealed a positive staining in all conditions. A significantly (p ​< ​0.001) higher synthesis of TNC within the B-L in comparison to the other two layers was observed (except for the scaffolds functionalised with TGF in T-L/TGF in FC-L, in which no significant (p ​> ​0.05) difference between the B-L and the T-L was observed). No significant (p ​> ​0.05) difference was observed between the synthesised amount of TNC in the B-L of non-functionalised and functionalised scaffolds (apart from the groups PDGF in T-L/BMP in FC-L and TGF in T-L/TGF in FC-L, where a significantly (p ​< ​0.05) lower amount was reported in the B-L compared to the control). No background staining was observed in acellular 3-L scaffolds.

### 3.5 Characterisation of functionalised 3-L scaffolds for chondrogenic differentiation

Immunohistochemistry and complementary image intensity analyses (Fig. 4b) at day 21 revealed that, among the BMs assessed in basal medium to induce hBMSC chondrogenic commitment, BMP-2, TGF-β3 and IGF-1 significantly (p ​< ​0.001) increased COL II and CS; and BMP-2, TGF-β3, IGF-1 and hyaluronic acid significantly (p ​< ​0.05) increased COL X. Consequently, BMP-2 and TGF-β3 were chosen as the most promising candidates for the FC-L functionalisation.

Cellular distribution (Fig. S6) analysis within the 3-L scaffolds functionalised for chondrogenic differentiation revealed a homogenous cell distribution throughout, at day 21.

**Fig. 6:**
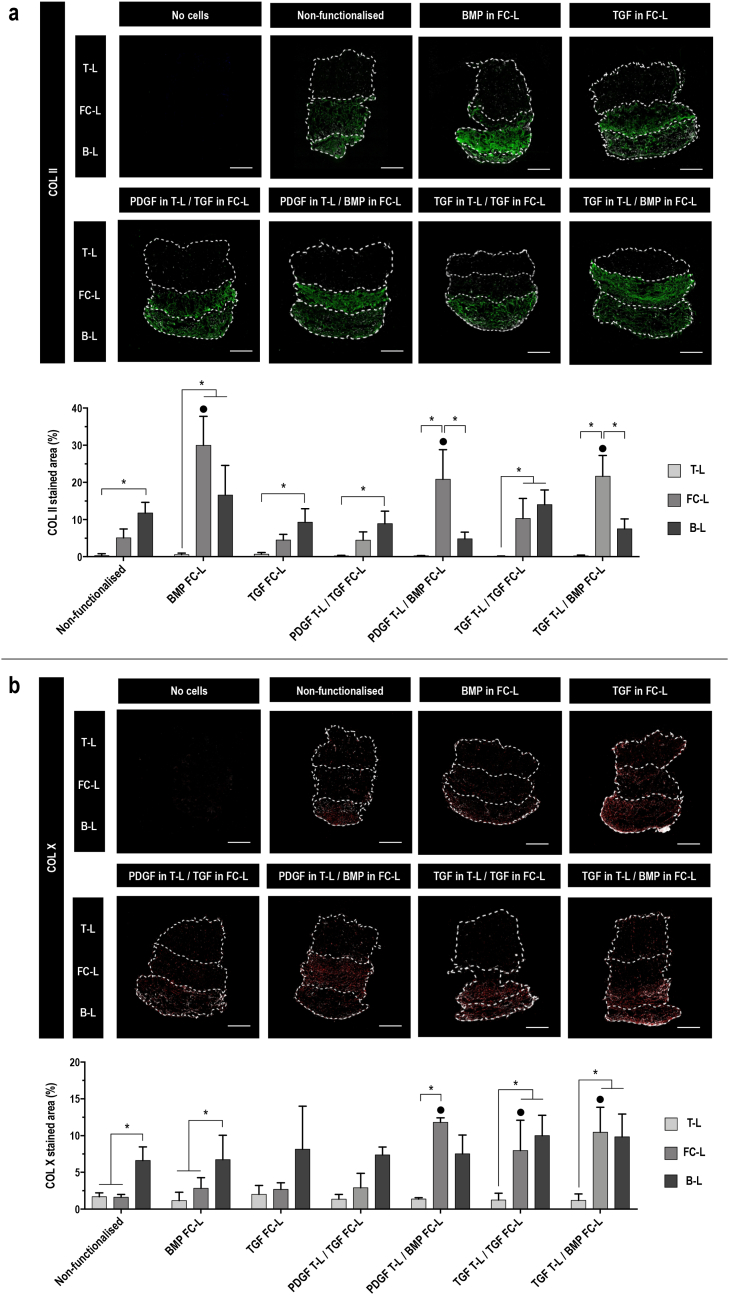
3-L functionalised scaffolds with BMP-2 promote a COL II and COL X deposition higher than non-functionalised scaffolds. **a**) COL II (green) and DAPI (white) fluorescent staining on paraffin slides and area quantification (%) at day 21 and **b**) COL X (red) and DAPI (white) fluorescent staining on paraffin slides and area quantification (%) at day 21 of acellular scaffolds (negative control) and hBMSCs cultured on 3-L scaffolds non-functionalised and functionalised with 1 ​GF in the FC-L (BMP in FC-L and TGF in FC-L) or 2 ​GFs in the T-L and FC-L (PDGF in T-L/TGF in FC-L, PDGF in T-L/BMP in FC-L, TGF in T-L/TGF in FC-L, TGF in T-L/BMP in FC-L); scale bar 500 ​μm. All data presented as mean ​± ​SD and N ​= ​3. ∗ indicates significant (p ​< ​0.05) difference between layers. • indicates significant (p ​< ​0.05) difference between FC-L of functionalised scaffolds vs FC-L of non-functionalised scaffolds. ▪□ indicates significant (p ​< ​0.05) difference between B-L of functionalised scaffolds vs B-L of non-functionalised scaffolds. (For interpretation of the references to colour in this figure legend, the reader is referred to the Web version of this article.)

Immunohistochemistry analysis of COL II at day 7 (Fig. S8a) of non-functionalised and functionalised 3-L scaffolds revealed a strong staining in all tested conditions, mainly localised in the FC-L. In the scaffolds functionalised with BMP in FC-L, TGF in FC-L, PDGF in T-L/BMP in FC-L and TGF in T-L/TGF in FC-L, the deposited COL II in the FC-L was significantly (p ​< ​0.05) higher than the remaining layers. Only in the scaffolds functionalised with BMP in FC-L, the COL II detected in the FC-L was significantly (p ​< ​0.05) higher than the FC-L of the control group. From the immunohistochemistry analysis of COL II at day 21 (Fig. 6a), a significantly (p ​< ​0.05) higher COL II deposition was detected in the FC-L functionalised with PDGF in T-L/BMP in FC-L and TGF in T-L/BMP in FC-L than the other layers for the scaffolds. COL II deposition was significantly (p ​< ​0.05) higher in the B-L than the T-L in the control, the TGF in FC-L and the PDGF in T-L/TGF in FC-L conditions; and COL II deposition was significantly (p ​< ​0.05) higher in the B-L and FC-L than the T-L of the scaffold functionalised with BMP in FC-L and TGF in T-L/TGF in FC-L scaffolds. All the scaffolds functionalised with BMP promoted a significantly (p ​< ​0.05) higher COL II synthesis in the FC-L than the control. No background staining was observed in acellular 3-L scaffolds from the porcine COL II forming the FC-L.

Immunohistochemistry analysis of COL X at day 7 (Fig. S8b) showed an overall fainted staining in all tested scaffolds. Immunohistochemistry analysis of COL X at day 21 (Fig. 6b) revealed a positive staining mostly localised in the FC-L and B-L regions of the scaffolds. FC-L and B-L equally promoted a significantly (p ​< ​0.05) higher synthesis of COL X than the T-L in the TGF in T-L/TGF in FC-L and TGF in T-L/BMP in FC-L scaffolds. The control and the BMP in FC-L scaffolds showed a significantly (p ​< ​0.05) higher synthesis of COL X in the B-L than the other layers. In the scaffolds functionalised with PDGF in T-L/BMP in FC-L, TGF in T-L/TGF in FC-L and TGF in T-L/BMP in FC-L, COL X synthesis was significantly (p ​< ​0.01) higher in the FC-L in comparison to the control. No background staining was observed in acellular 3-L scaffolds.

### 3.6 *Ex vivo* assessment of the 3-L collagen scaffolds

**Fig. 7:**
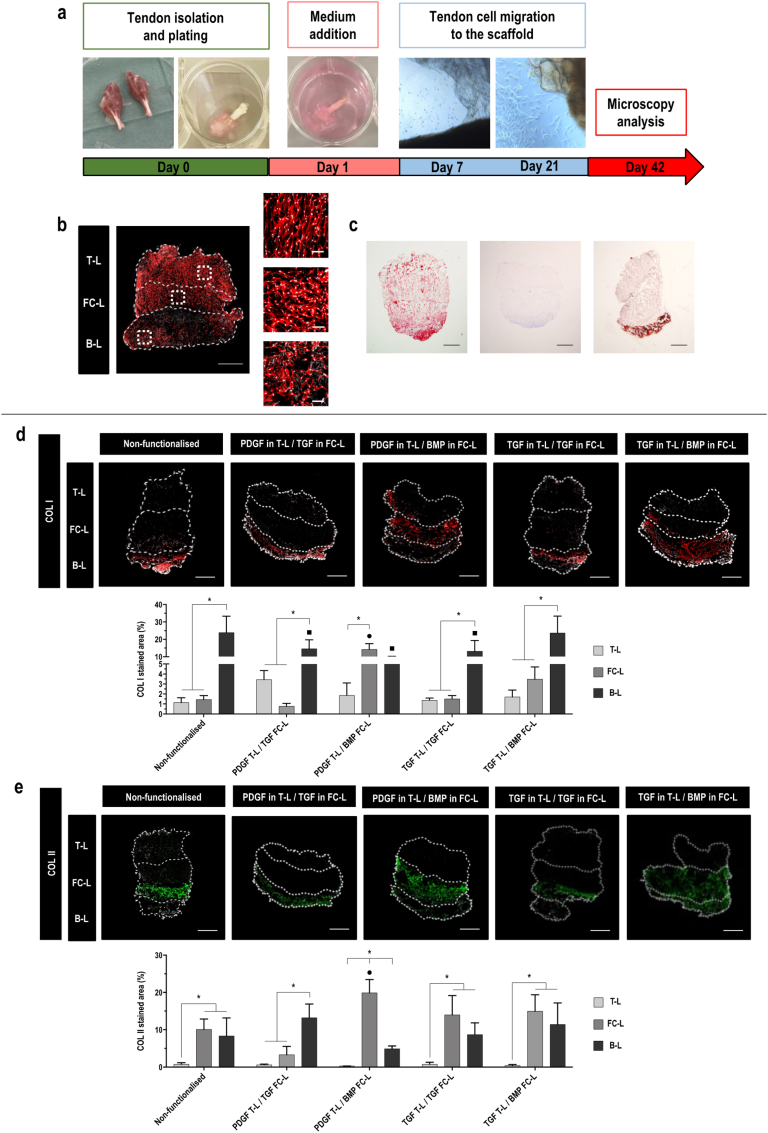
3-L non-functionalised scaffolds support the growth of tendon - derived cells and promote COL II deposition in an *ex vivo* model. **a**) Achilles tendon organotypic culture experimental timeline from day 0 to day 42. **b**) Rhodamine labelled phalloidin (red) and DAPI (white) staining at day 42 of tendon – derived cells migrated into 3-L non-functionalised scaffolds in basal medium; 3-L scaffold (scale bar 1 ​mm) and magnification of the T-L, FC-L and B-L (scale bar 100 ​μm). **c**) Picrosirius red, alcian blue and alizarin red staining at day 42 of tendon - derived cells migrated into 3-L non-functionalised scaffolds; scale bar 2 ​mm. **d**) COL I (red) and DAPI (white) fluorescent staining on paraffin slides and area quantification (%) at day 42 and **e**) COL II (green) and DAPI (white) fluorescent staining on paraffin slides and area quantification (%) at day 42 of tendon - derived cells migrated into 3-L scaffolds non-functionalised and functionalised with 2 ​GFs in the T-L and FC-L (PDGF in T-L/TGF in FC-L, PDGF in T-L/BMP in FC-L, TGF in T-L/TGF in FC-L, TGF in T-L/BMP in FC-L); scale bar 500 ​μm. All data presented as mean ​± ​SD and N ​= ​3. ∗ indicates significant (p ​< ​0.05) difference between layers. • indicates significant (p ​< ​0.05) difference between FC-L of functionalised scaffolds vs FC-L of non-functionalised scaffolds. ▪□ indicates significant (p ​< ​0.05) difference between B-L of functionalised scaffolds vs B-L of non-functionalised scaffolds. (For interpretation of the references to colour in this figure legend, the reader is referred to the Web version of this article.)

Microscopic (Fig. 7a) observation of Achilles’ tendon explants from adult rats plated into well plates next to 3-L scaffolds showed that tendon -derived cells migrated outside the tendon explants and reached confluency after 21 days. Qualitative morphological (Fig. 7b) analysis after 21 more days revealed that cells spread throughout the 3-L scaffolds, preferentially along the borders, appearing with an elongated cytoskeleton in the T-L.

Qualitative histological (Fig. 7c) analysis revealed a homogeneous presence of total collagen throughout the scaffold (although more dominant in the B-L), almost absent GAG deposition and a strong calcium content in the B-L of the scaffolds.

COL I immunohistochemistry (Fig. 7d) analysis on non-functionalised and double-functionalised 3-L scaffolds after 21 days of culture exhibited a significant (p ​< ​0.05) increase of the stained area in the B-L compared to the other two layers in all tested conditions, except for the scaffolds functionalised with PDGF in T-L/BMP in FC-L, in which there was no significant (p ​> ​0.05) difference between the B-L and the FC-L. No significant (p ​> ​0.05) difference was observed between the amount of COL I synthesised in the B-L of non-functionalised scaffolds and the B-L of TGF in T-L/BMP in FC-L scaffolds, whilst in all the other groups it was significantly (p ​< ​0.05) lower.

Immunohistochemistry (Fig. 7e) analysis of COL II on non-functionalised and double-functionalised 3-L scaffolds at day 21 revealed a significantly (p ​< ​0.05) higher staining in the FC-L and B-L than the T-L in the control, the TGF in T-L/TGF in FC-L and TGF in T-L/BMP in FC-L scaffolds. The COL II amount was significantly (p ​< ​0.05) higher in the FC-L than the other layers of the PDGF in T-L/BMP in FC-L scaffolds and in the B-L than the other layers of the PDGF in T-L/TGF in FC-L scaffolds. No significant (p ​> ​0.05) difference between the deposited COL II in the FC-L of the functionalised scaffolds and the control scaffolds was detected, except for the PDGF in T-L/BMP in FC-L scaffolds, where it was significantly (p ​< ​0.05) higher.

## 4 Discussion

Functional enthesis regeneration remains an open clinical challenge as the traditional surgical reattachment of the tendon directly to bone tissue impairs the native tissue properties by leaving behind a scar populated by fibroblasts. In this study, we developed a 3-L bioinspired, with respect to native tissue composition, scaffold that mimics the collagenous composition of the enthesis and we enhanced its biological potential by spatial incorporation of BMs, to drive hBMSC differentiation towards the relevant cell populations of the enthesis. The developed 3-L scaffold was validated *in vitro* using hBMSCs and in an *ex vivo* model.

### 4.1 Physicochemical characterisation of non-functionalised scaffolds

Although the intrinsic complexity of the enthesis calls for sophisticated constructs, many of the proposed works are burdened by either a complex composition or low technical and manufacturing feasibility, which can inevitably raise not only the synthesis and fabrication costs, but also the number of quality-control steps in a clinically relevant setting [[41], [42], [43], [44]]. In this work, 3-L scaffolds were fabricated with COL I and COL II extracted from a single animal source (porcine), with the addition of osteoconductive hydroxyapatite particles, largely and safely used to reinforce collagenous matrices [56,57], to facilitate regulatory clearance in a clinical scenario. The three collagenous layers were iteratively frozen and the final construct was processed in a freeze dryer in one single step. This is of importance, considering that similar collagen-based layered scaffolds have been fabricated with multiple freeze drying, hydration and cross-linking steps [26,37,58], which can jeopardise the clinical approval process. The scaffolds presented a triphasic yet continuous structure and a network of interconnected pores with different morphology: large and elongated in the T-L and FC-L and small and round in the B-L, as a consequence of the freeze drying process, during which the pore structure obtained is a negative replica of the ice crystals morphology after freezing [59,60].

Assessment of the cross-linking efficacy showed that the use of PEG-SG significantly improved the physicochemical properties and enzymatic degradation rate of the single layers and the full construct, as previously well documented [48,61], compared to the non-cross-linked scaffolds. The superior resistance of the B-L (in comparison to the T-L and FC-L) to enzymatic degradation can be explained by a potential competition of the HAp to the collagenase cleavage sites, or by the absorption of some of the enzyme to the surface of the HAp [62]. The strengthening effect of HAp also manifested in the highest ultimate compressive strength values reported for the 3-L scaffold and the B-L, compared to the other two layers, attributed to a mechanical load transfer from the collagen matrix to the rigid apatite crystals [63].

### 4.2 *In vitro* characterisation of non-functionalised scaffolds

Cytocompatibility analysis showed that the 3-L scaffolds uniformly supported hBMSC growth; the highest metabolic activity was registered at day 3, likely as a consequence of a first adaptation time required by the cells post-seeding procedure, while it decreased over time, indicative of active cell differentiation taking place [64]. From the cytoskeleton staining was evident a certain degree of alignment for the cells within the T-L, which had a peak at day 7 and then decreased. This could be explained considering that cells are initially more capable of sensing the architectural guidance of the scaffold by contact guidance [65], while over time, the cell-cell contact becomes the predominant driver of cellular alignment [66]. Although we only achieved a certain amount of aligned pores in the upper region of the T-L and FC-L, this can be already beneficial for the initial differentiation of BMSCs. Indeed, studies have proven that an anisotropically oriented matrix can aid cell commitment towards tenogenic [67,68] and chondrogenic lineages [69,70], by resembling the ECM of tendon tissue and the superficial zone of the fibrocartilage and articular cartilage, respectively.

With respect to the tenogenic potential of the 3-L scaffolds, the picrosirius red staining showed that the total collagen (which we can assume was primarily COL I, since it is abundantly synthesised by all the enthesis cell populations [71]) was deposited homogeneously throughout the scaffold in basal and differentiation media. The Alcian blue staining detected the presence of GAGs locally distributed within the FC-L, but only when chondrogenic medium was used. This is indicative of the fact that, although the sole scaffold porous architecture and collagenous composition were not enough to elicit a response, the combined effect with the differentiation medium was able to drive hBMSC differentiation toward a fibrocartilage cell lineage in a regional specific fashion. Finally, the copious presence of calcium and high activity of ALP in the B-L, pointed out the potential of the scaffold to drive the differentiation of hBMSCs towards the osteogenic lineage in a spatial specific manner, even without exposition to the osteogenic medium. This is most certainly due to the presence of HAp, a known osteogenesis inducer [57], within the B-L and to the increased stiffness of this layer [72]. Overall, these results remark the suitability of the fabrication approach to promote and maintain a regional compartmentalisation of the mineral content, which is highly advocated to not hinder the slower fibrocartilage and tendon repair, in an *in vivo* setting and to initiate the correct healing process. Indeed, it has been shown that poor enthesis repair is also related to mineral loss of the existing bone tissue, which starts after the tear of the tendon, possibly because of a loss in mechanical loading [7] and after surgical intervention, caused by increased activity of osteoclast cells in the adjacent bone [73].

Generally, the absence of layer-specificity in the gene expression profile could be due to the visual approximation of the layer borders or to the overshadowing influence of the soluble factors of the differentiation media over the scaffold architectural cues. When scaffolds were cultured in basal medium instead, data obtained can be attributed to the paracrine effect of soluble factors released by hBMSCs, as previously documented [[74], [75], [76]]. Nonetheless, the robustness of tenogenic, chondrogenic and osteogenic differentiation was reflected by the progressive increases in mRNAs respect to cells cultured on TCP for some of the most known markers. Specifically, the 3-L scaffolds promoted the upregulation of tendon markers, such as TNMD, SCXA, COL1A1 and COL3A1 and TNC, in both basal and tenogenic media, compared to cells cultured on TCP. MKX was the only downregulated gene, which could be attributed to a negative feedback loop phenomenon during which accumulation of a protein induces cells to suppress the expression of the corresponding gene [77]. The gene expression profile for chondrogenic markers, such as COL2A1 and COL10A1 and SOX9, confirmed the role of the differentiation medium in driving hBMSC differentiation, although not in a zonal specific fashion. Of note, also cells cultured on 3-L scaffolds in basal medium reported a higher level of SOX9 respect to cell on TCP, pointing out the critical role of the COL II scaffold composition on cell differentiation. In both basal and differentiation media, the tendency of hBMSCs towards the osteochondral ossification [78] can explain the early upregulation of COL10A1, which is a key component of mineralised fibrocartilage. Regarding the osteogenic expression profile, all the tested genes such as RUNX 2, BGLAP and SPP1 were upregulated in basal medium in the B-L at day 7, whilst in osteogenic medium, they were upregulated at day 21, except for the downregulated BGLAP, which could be again attributed to negative feedback loop phenomena.

### 4.3 *In vitro* characterisation of functionalised scaffolds

To enhance the spatial hBMSC differentiation towards the tendon and the fibrocartilage lineages, we decided to explore a zonal functionalisation of the scaffold with BMs. BMs, in particular growth factors (GFs), are progressively emerging as a powerful tool to improve healing outcomes in the tendon-to-bone interface [79,80]. Given the multiphasic nature of the enthesis, combinations or gradients of BMs are likely to be needed to provide the seeded cell populations with differential stimuli [32]. To this end, a number of multi-domain/multi-cargo delivery vehicles have been successfully developed in recent years to achieve a controlled, spatial and temporal release of the loaded molecules [36]. When it comes to the functionalisation of ECM-derived materials, BMs have been loaded either by direct incorporation or by covalent and non-covalent immobilisation [81]. This last strategy can be performed by physical entrapment of the molecules within the not-yet formed carrier, affinity based binding or ionic complexation [82]; overall, it offers a higher control over the release compared to the direct molecule incorporation into the pre-formed vehicle (by manipulation of the properties of the carrier, such as porosity, pore size, degree of crosslinking and degradation rate [83]) and does not share the molecule bioactivity and orientation concerns of the covalent-immobilisation [84]. Considering all the pros and cons, we decided to perform a scaffold functionalisation that would increase the off-shelf potential of the device while still preserving the cargo bioactivity. Precisely, the chosen molecules were loaded in the collagen solution forming the T-L and the FC-L before the freeze drying process, since the biological activity of different GFs, including PDGF-bb, BMP-2 and TGF-β1, has already been showed to be preserved during this procedure [31,85]. After an initial screening, we selected PDGF-bb and TGF-β3 to be functionalised within the T-L, and BMP-2 and TGF-β3 to be functionalised within the FC-L, as these are some of the key GF contributors at the site of injury by enhancing the healing response through cell recruitment, proliferation, ECM synthesis and remodelling at the repair site [80]. When we evaluated COL I and TNC deposition to assess the effect of the T-L functionalisation, both marker levels were higher in the B-L compared to the other two layers, in all tested groups. This is in agreement with previous publications, where TNC has been associated with early stages chondrogenesis [86,87] and osteogenesis [88,89]. Regarding the potential of functionalised molecules in the FC-L, the single and double functionalisation with BMP-2 greatly improved chondrogenic differentiation in a local manner within the FC-L, by increasing COL II and COL X synthesis. A possible explanation as why none of the tested BMs had a distinctive action in the synthesis of ECM components when loaded in the T-L, could be the weaker structure of this individual region (since it had lower ultimate compressive strength values than the 3-L scaffolds) and the presence of bigger pores which lead to a faster release of the entrapped BMs [90]. Although comparable in terms of pore size to the T-L, the FC-L is not as exposed (as the T-L or the B-L) to the medium present matrix metalloproteinases [91] thanks to its protected middle layer position, which could have possibly given more time to the encapsulated BMs to exert their action. Additionally, since BMP-2 and the members of the TGF-β family have a natural affinity for collagen, we can speculate a chemoprotective role of these molecules by the scaffold [[92], [93], [94], [95]], as well as by the bonding to the PEG [96].

### 4.4 *Ex vivo* characterisation of the non-functionalised and functionalised scaffolds

*Ex vivo* explant models enable insights that cannot be gained using *in vitro* models, since they maintain the original cell sub-populations within their native organisation and ECM composition; they also offer a more practical and economical tool than the *in vivo* models to study in a controlled way different biological variables [97]. As a proof of principle of the findings obtained from the *in vitro* model and to reproduce an *in vivo* post-implantation scenario, we exploited a rat Achilles tendon explant model to assess whether native tendon – derived cells were able to migrate and proliferate within the 3-L scaffolds and along which cell lineage they would differentiate, in non-functionalised and functionalised scaffolds. Starting with morphological analysis, cells were observed to have spread uniformly across the scaffold, although more abundantly along the peripherical area, which might imply that a longer time is required for a complete cell migration into the core of the construct. A bidirectional cell orientation was seen in T-L, which confirmed the propensity of the cells to align following the pore morphology of this layer. Histological characterisation of the 3-L scaffolds in basal medium highlighted similar findings of the *in vitro* model, such as an abundant presence of total collagen throughout the scaffold, but more predominantly in the B-L; low GAG deposition, also localised in the B-L; and a strong and confined presence of calcium in the B-L. Taken together, these results corroborate the role of the sole scaffold composition and architecture in driving differentiation of native tendon cells towards an osteogenic phenotype and the need for a combinatorial approach with BMs to boost tenogenic and chondrogenic differentiation. When scaffolds were stained for COL I, non-functionalised constructs showed an intense staining in the B-L, which was as high as in the TGF in T-L/BMP in FC-L scaffolds and greater than the other functionalised scaffolds. Moving to the COL II staining, the non-functionalised scaffolds showed a quite intense deposition localised in the FC-L and the B-L, which was as intense as the levels reached by the functionalised scaffolds and only surpassed by the scaffolds functionalised with PDGF in T-L/BMP in FC-L. Many *in vivo* studies have highlighted the pivotal role of the fibrocartilage for a functional enthesis repair, since it enhances the strength of the reattachment between bone and repaired tendon [11,98]. Although preliminary, this is an encouraging result, as it means that the native tendon-derived cells could be able to differentiate towards a fibrochondrogenic population and initiate the tissue repair, in an *in vivo* scenario. Regarding the absence of a stronger outcome from the GF functionalisation, this could be to the fact that the loaded BMs have been progressively washed out from the scaffold during the first days in culture, when the cells were migrating outside of the tendon explants. If, on one side, this might have helped native cells to migrate in the scaffolds, as GFs have been shown to be potent chemoattractants [99], the fact that also the non-functionalised scaffolds were fully and homogeneously invaded by cells, points out the potential of the scaffold alone to sustain repopulation in an *in vivo* setting. This is of relevance, as several studies had to resort to the combined aid of bioactive molecules [100,101] to have the same effect and because it is not uncommon for these kind of constructs to have issues of repopulation after implantation [102]. Future studies could include a single molecule functionalised within the T-L, as it is likely that the FC-L does not need any additional functionalisation. We recognise also that the mechanical properties of the 3-L constructs do not match the original stiffness of the fibrocartilage, however many studies have shown that initial MSC chondrogenic commitment can benefit from softer materials, since they undergo dimensional contraction, which in turn promote cellular condensation and the subsequent chondrogenic differentiation [[103], [104], [105]]. On a similar note, while the stiffness of mature tendon is in the order of hundreds of MPa, the healing tendon matrix is considerably softer, reporting values in the order of few kPa [106]. Additionally, some works have showed that low stiffness collagen substrates can elicit a stronger tenogenic response than stiffer ones [107]. However, a slight increase of the T-L stiffness could be beneficial to retain the loaded molecules longer and more efficiently, boosting tenogenic differentiation. Finally, a relevant *in vivo* model would be needed to substantiate the findings of this work.

## 5 Conclusions

Our work offers new insights on the combinational outcomes of scaffold collagenous composition, porous architecture, and loaded GFs on the regulation of hBMSC differentiation towards the relevant cell populations of the enthesis. In particular, we demonstrated that BMP-2 functionalised 3-L scaffolds were able to promote local differentiation of cells towards the osteogenic and fibrochondrogenic lineages *in vitro*. These findings were also corroborated by an *ex vivo* model, in which tendon - derived cells migrated into the 3-L scaffolds and initiated, even in absence of GF functionalisation, the formation of an osteo/fibrocartilage interface, which can be beneficial to strengthen the attachment between the existing bone and the repaired tendon. Future improvements of the 3-L scaffolds will include a stronger cell commitment towards the tenogenic lineage to fully recapitulate the graded structure of the enthesis. Although the quest for the optimal 3-L scaffold is still ongoing, we believe this work holds the promise for an *in vivo* functional repair of the tendon-to-bone interface.

## Credit author statement

Eugenia Pugliese: Conceptualised, Writing – original draft, Data curation, Writing – review & editing, Formal analysis, Investigation, Methodology. Ignacio Sallent Cucurella: Formal analysis, Data curation, Investigation, Methodology. Sofia Ribeiro: Formal analysis, Data curation, Investigation, Methodology. Alexandre Trotier: Formal analysis, Data curation, Investigation, Methodology. Stefanie H. Korntner: Formal analysis, Data curation, Investigation, Methodology. Yves Bayon: Supervision, Writing – review & editing. Dimitrios I. Zeugolis: Supervision, Funding acquisition, Resources, Project administration, Conceptualised, Writing – original draft, Writing – review & editing.

## Declaration of competing interest

The authors declare the following financial interests/personal relationships which may be considered as potential competing interests: Yves Bayon is an employee of Medtronic, France. The other authors (Eugenia Pugliese, Ignacio Sallent, Sofia Ribeiro, Alexandre Trotier, Stefanie H. Korntner, Dimitrios I. Zeugolis) declare no competing interests.

## Data Availability

Data will be made available on request.
