# Supplementary material for: Development of three-layer collagen scaffolds to spatially direct tissue-specific cell differentiation for enthesis repair
Source: Mater Today Bio. 2023 Mar 7;19:100584. doi: 10.1016/j.mtbio.2023.100584 (PMC10034511; doi:10.1016/j.mtbio.2023.100584)
Supplement: Multimedia component 1 [file mmc1.docx]

# Title

Development of three-layer collagen scaffolds to spatially direct tissue-specific cell differentiation for enthesis repair

# Authors

Eugenia Pugliese (1, 2), Ignacio Sallent (1, 2), Sofia Ribeiro (1, 3), Alexandre Trotier (2), Stefanie H. Korntner (1, 2), Yves Bayon (3), Dimitrios I. Zeugolis* (1, 2, 4)

# Affiliations

1. Regenerative, Modular & Developmental Engineering Laboratory (REMODEL), University of Galway, Galway, Ireland

2. Science Foundation Ireland (SFI) Centre for Research in Medical Devices (CÚRAM), University of Galway, Ireland

3. Medtronic, Sofradim Production, Trevoux, France

4. Regenerative, Modular & Developmental Engineering Laboratory (REMODEL), Charles Institute of Dermatology, Conway Institute of Biomolecular & Biomedical Research and School of Mechanical & Materials Engineering, University College Dublin (UCD), Dublin, Ireland

* Corresponding Author: Dimitrios I. Zeugolis, REMODEL, UCD. Telephone: +353 (0) 1716 1887. Email: dimitrios.zevgolis@ucd.ie

**Supplementary Table S1:** TaqMan primer probe assay IDs used for gene analysis.

| **Marker** | **Gene Name** | **Gene Symbol** | **Assay ID** |
| --- | --- | --- | --- |
| Tenogenic | Collagen I | COL1A1 | Hs.PT.56a.40971026 |
|  | Scleraxis homolog A | SCXA | Hs03054634_g1 |
|  | Tenascin C | TNC | Hs.PT.58.2529606 |
|  | Tenomodulin | TNMD | Hs.PT.58.14567828 |
|  | Collagen III | COL3A1 | Hs.PT.58.4249241 |
|  | Mohawk | MKX | Hs.PT.58.40174404 |
| Osteogenic | Runt-related transcription factor | RUNX2 | Hs.PT.56a.19568141 |
|  | Secreted phosphoprotein 1 | SPP1 | Hs.PT.58.19252426 |
|  | Bone gamma-carboxyglutamate protein | BGLAP | Hs.PT.56a.39318706.g |
| Chondrogenic | Collagen II | COL2A1 | Hs.PT.58.39833355 |
|  | Collagen X | COL10A1 | Hs.PT.58.38672730 |
|  | SRY-Box Transcription Factor 9 | SOX9 | Hs.PT.58.38984663 |
| Reference | Glyceraldehyde-3-phosphate dehydrogenase | GAPDH | Hs.PT.39a.22214836 |
|  | 60S acidic ribosomal protein P0 | RPLP0 | Hs.PT.56.40434846 |
|  | β2 microglobulin | B2M | Hs.PT.58v.18759587 |

**Supplementary Table S2:** Tested bioactive molecules, concentrations and rational of choice.

| **Molecule** | **Catalogue number** | **Supplier** | **Type of functionalisation** | **Concentration** | **Rational** |
| --- | --- | --- | --- | --- | --- |
| PDGF-bb | 100-14B | PeproTech, France | Supplemented in medium for tenogenic differentiation | 20 ng/ml | Upregulated in the early phases of the tendon healing process, it stimulates cell migration, proliferation and matrix deposition [1] |
|  |  |  | Functionalised in T-L | 100 ng/ml |  |
| TGF-β3 | 8420-B3 | BioTechne, USA | Supplemented in medium for tenogenic differentiation | 20 ng/ml | Associated with scar-free healing, it promotes chondrogenic [2] and tenogenic [3] differentiation of MSCs |
|  |  |  | Functionalised in T-L | 100 ng/ml |  |
|  |  |  | Supplemented in medium for chondrogenic differentiation | 10 ng/ml |  |
|  |  |  | Functionalised in FC-L | 50 ng/ml |  |
| BMP-2 | H4791 | Sigma-Aldrich, Ireland | Supplemented in medium for chondrogenic differentiation | 100 ng/ml | Osteoinductive factor involved in osteogenic and chondrogenic differentiation (through proteoglycans and COL II synthesis) [4] |
|  |  |  | Functionalised in FC-L | 300 ng/ml |  |
| IGF-1 | 291-G1 | BioTechne, USA | Supplemented in medium for tenogenic differentiation | 20 ng/ml | Modulates macrophage polarisation to promote inflammation resolution in tendon and muscle injuries [5] besides being able to promote osteogenesis and chondrogenesis of BMSCs [6] |
|  |  |  | Supplemented in medium for chondrogenic differentiation | 100 ng/ml |  |
| GDF-7 | SRP4572 | Sigma-Aldrich, Ireland | Supplemented in medium for tenogenic differentiation | 100 ng/ml | Expressed in developing tendons (promotes tendon specific differentiation by expressing tenascin, scleraxis, tenomodulin, fibronectin and collagen type III and I) [7] |
| GDF-5 | 8340-G5 | BioTechne, USA | Supplemented in medium for tenogenic differentiation | 100 ng/ml | Expressed in developing tendons [8] but also associated with fibrocartilage differentiation (through the hedgehog pathway) [9] |
| HYA | 53747 | Sigma-Aldrich, Ireland | Supplemented in medium for chondrogenic differentiation | 0.05% w/v | A glycosaminoglycan and an important component of the cartilage, supports chondrocyte matrix deposition and chondrogenic differentiation of MSCs [10] |
| KGN | SML0370 | Sigma-Aldrich, Ireland | Supplemented in medium for chondrogenic differentiation | 10 μM | A drug-like molecule that promotes chondrogenic differentiation by increasing expression of collagen type II and aggrecan [11] |

**Supplementary Table S3:** List of primary and secondary antibodies used for immunohistochemistry.

| **Antibody** | **Catalogue number** | **Supplier** | **Reactivity** | **Dilution** |
| --- | --- | --- | --- | --- |
| Collagen type I | PA2140 | BosterBio, USA | Rabbit anti-human | 1:200 |
| Collagen type III | ab7778 | Abcam, UK | Rabbit anti-human | 1:200 |
| Tenascin | ab88280 | Abcam, UK | Mouse-anti-human | 1:200 |
| Collagen type II | ab185430 | Abcam, UK | Mouse-anti-human | 1:200 |
| Collagen type X | ab58632 | Abcam, UK | Rabbit anti-human | 1:200 |
| Chondroitin sulphate | C8035 | Sigma-Aldrich, Ireland | Mouse-anti-human | 1:150 |
| Anti-mouse 488 | A-21202 | Invitrogen, Ireland | Donkey anti-human | 1:400 |
| Anti-Rabbit 594 | A-21207 | Invitrogen, Ireland | Donkey anti-human | 1:400 |

**Figure S1:** Characterisation of extracted COL I, COL II and COL I / HAp suspension. **a**) SDS-PAGE of commercially available (standard) vs in-house extracted COL I (left) and COL II (right). **b**) EDX spectrum and quantification of carbon, nitrogen, oxygen and gold of in-house extracted COL I (left) and COL II (right). **c**) EDX spectrum; quantification of carbon, nitrogen, oxygen, calcium, phosphorus and gold; elemental mapping of phosphorus (green), calcium (orange) and merge; and FT-IR spectra of COL I, HAp and COL I / HAp suspension.

**Figure S2:** Picrosirius red and alizarin red staining on acellular scaffolds. **a**) Picrosirius red staining on paraffin slides of acellular 3-L scaffolds in basal medium at day 21; scale bar 2 mm. **b**) Alizarin red staining on paraffin slides of acellular 3-L scaffolds in basal medium at day 21; scale bar 2 mm.

**Figure S3:** GAGs quantification of 3-L scaffolds cultured in basal and chondrogenic media. Total amount of GAGs normalised by DNA performed with DMMB assay of hBMSCs in the T-L, FC-L and B-L of 3-L scaffolds cultured in basal and chondrogenic media at day 7 and day 21. All data presented as mean ± SD and N=3. * indicates significant (p < 0.05) difference between layers.

**Figure S4:** ALP activity and calcium quantification analyses of 3-L scaffolds cultured in basal and osteogenic media. **a**) ALP activity normalised by DNA (pmol/ min / μg/ml) of hBMSCs in the T-L, FC-L and B-L of 3-L scaffolds cultured in basal and osteogenic media, at day 7 and 21. **b**) Amount of calcium normalised by DNA of hBMSCs in the T-L, FC-L and B-L of 3-L scaffolds cultured in basal and osteogenic media, at day 7 and 21. All data presented as mean ± SD and N=3. * indicates significant (p < 0.05) difference between layers.

**Figure S5:** Gene profile expression of hBMSCs cultured on 3-L scaffolds in basal and differentiation media. **a**) Tenogenic, **b**) chondrogenic and **c**) osteogenic gene expression profile of hBMSCs cultured on 3-L scaffolds in basal and differentiation media, assessed by TaqMan^®^ array at day 7 and 21. Data are expressed in relation to cells cultured on TCP at a given time point. 3 biological replicates were analysed by pulling together 6 technical replicates. Grey background, not detectable; white background unchanged; green background, two-fold downregulated; red background, two-fold upregulated.

**Figure S6:** Cell distribution analysis of hBMSCs on non-functionalised and functionalised 3-L scaffolds. Cytoskeleton of cells stained with rhodamine labelled phalloidin (red) and nuclei with DAPI (white) of hBMSCs seeded on 3-L scaffolds non-functionalised and functionalised with 1 GF in the T-L (PDGF in T-L and TGF in T-L) and FC-L (BMP in FC-L and TGF in FC-L) or 2 GFs in the T-L and FC-L (PDGF in T-L / TGF in FC-L, PDGF in T-L / BMP in FC-L, TGF in T-L / TGF in FC-L, TGF in T-L / BMP in FC-L) after 21 days in culture; scale bar 500 µm. On the bottom, quantification of rhodamine labelled phalloidin stained area (%). All data presented as mean ± SD and N=3.

**Figure S7:** hBMSC deposited COL I and TNC on functionalised 3-L scaffolds. **a**) COL I (red) and DAPI (white) fluorescent staining on paraffin slides and area quantification (%) at day 7 and **b**) TNC (green) and DAPI (white) fluorescent staining on paraffin slides and area quantification (%) at day 7 of hBMSCs cultured on 3-L scaffolds non-functionalised and functionalised with 1 GF in the T-L (PDGF in T-L and TGF in T-L) or 2 GFs in the T-L and FC-L (PDGF in T-L / TGF in FC-L, PDGF in T-L / BMP in FC-L, TGF in T-L / TGF in FC-L, TGF in T-L / BMP in FC-L); scale bar 500 µm. All data presented as mean ± SD and N=3. * indicates significant (p < 0.05) difference between layers. ◼︎ indicates significant (p < 0.05) difference between B-L of functionalised scaffolds vs B-L of non-functionalised scaffolds.

**Figure S8:** hBMSC deposited COL II and COL X on functionalised 3-L scaffolds. **a**) COL II (green) and DAPI (white) fluorescent staining on paraffin slides and area quantification (%) at day 7 and **b**) COL X (red) and DAPI (white) fluorescent staining on paraffin slides and area quantification (%) at day 7 of hBMSCs cultured on 3-L scaffolds non-functionalised and functionalised with 1 GF in the FC-L (BMP in FC-L and TGF in FC-L) or 2 GFs in the T-L and FC-L (PDGF in T-L / TGF in FC-L, PDGF in T-L / BMP in FC-L, TGF in T-L / TGF in FC-L, TGF in T-L / BMP in FC-L); scale bar 500 µm. All data presented as mean ± SD and N=3. * indicates significant (p < 0.05) difference between layers. ● indicates significant (p < 0.05) difference between FC-L of functionalised scaffolds vs FC-L of non-functionalised scaffolds. ◼︎ indicates significant (p < 0.05) difference between B-L of functionalised scaffolds vs B-L of non-functionalised scaffolds.

# References

[1] O. Evrova, J. Buschmann, In vitro and in vivo effects of PDGF-BB delivery strategies on tendon healing: A review, Eur Cell Mater 34 (2017) 15-39.

[2] K. Brady, S.C. Dickinson, P.V. Guillot, J. Polak, A.W. Blom, W. Kafienah, A.P. Hollander, Human fetal and adult bone marrow-derived mesenchymal stem cells use different signaling pathways for the initiation of chondrogenesis, Stem Cells Dev 23(5) (2014) 541-554.

[3] M. Leung, S. Jana, C.T. Tsao, M. Zhang, Tenogenic differentiation of human bone marrow stem cells via a combinatory effect of aligned chitosan-poly-caprolactone nanofibers and TGF-beta3, J Mater Chem B 1(47) (2013) 6516-6524.

[4] J.E. Samorezov, E. Alsberg, Spatial regulation of controlled bioactive factor delivery for bone tissue engineering, Adv Drug Deliv Rev 84 (2015) 45-67.

[5] F. Mourkioti, N. Rosenthal, IGF-1, inflammation and stem cells: Interactions during muscle regeneration, Trends Immunol 26(10) (2005) 535-542.

[6] A. Youssef, D. Aboalola, V.K. Han, The roles of insulin-like growth factors in mesenchymal stem cell niche, Stem Cells Int 2017 (2017) 9453108.

[7] S.C. Fu, Y.P. Wong, B.P. Chan, H.M. Pau, Y.C. Cheuk, K.M. Lee, K.-M. Chan, The roles of bone morphogenetic protein (BMP) 12 in stimulating the proliferation and matrix production of human patellar tendon fibroblasts, Life Sci 72(26) (2003) 2965-2974.

[8] H. Aslan, N. Kimelman-Bleich, G. Pelled, D. Gazit, Molecular targets for tendon neoformation, J Clin Invest 118(2) (2008) 439-444.

[9] N.A. Dyment, A.P. Breidenbach, A.G. Schwartz, R.P. Russell, L. Aschbacher-Smith, H. Liu, Y. Hagiwara, R. Jiang, S. Thomopoulos, D.L. Butler, D.W. Rowe, Gdf5 progenitors give rise to fibrocartilage cells that mineralize via hedgehog signaling to form the zonal enthesis, Dev Biol 405(1) (2015) 96-107.

[10] I.L. Kim, R.L. Mauck, J.A. Burdick, Hydrogel design for cartilage tissue engineering: A case study with hyaluronic acid, Biomaterials 32(34) (2011) 8771-8782.

[11] K. Johnson, S. Zhu, M.S. Tremblay, J.N. Payette, J. Wang, L.C. Bouchez, S. Meeusen, A. Althage, C.Y. Cho, X. Wu, P.G. Schultz, A stem cell-based approach to cartilage repair, Science 336(6082) (2012) 717-721.
